# Stratification system for pharmaceutical care in cancer patients: Chinese expert consensus

**DOI:** 10.3389/fphar.2025.1707229

**Published:** 2026-02-16

**Authors:** Ya Chen, Xue Ma, Mao Lin, Zhiqiang Hu, Junxiang Zhou, Yue Qiu, Zhixi Liu, Suya Du, Lihong Shi, Li Han, Yan Chen, Tingting Qi, Yu Zhang, Guohui Li, Hongtao Xiao

**Affiliations:** 1 Department of Pharmacy, Sichuan Clinical Research Center for Cancer, Sichuan Cancer Hospital & Institute, Sichuan Cancer Center, Affiliated Cancer Hospital of the University of Electronic Science and Technology of China, Chengdu, China; 2 Department of Pharmacy, Union Hospital, Tongji Medical College, Huazhong University of Science and Technology, Wuhan, China; 3 Department of Pharmacy, National Cancer Center, National Clinical Research Center for Cancer, Cancer Hospital, Chinese Academy of Medical Sciences and Peking Union Medical College, Beijing, China

**Keywords:** cancer patients, Chinese, expert consensus, pharmaceutical care, stratification system

## Abstract

**Background:**

China bears one of the world’s heaviest cancer burdens, with a complex and multimodal cancer treatment environment. Currently, the absence of a standardized framework for oncology pharmaceutical care limits the quality and precision of services, highlighting an urgent need for clinical guidance.

**Objective:**

The objective of this study was to establish an evidence- and practice-informed consensus on oncology pharmaceutical care, providing a standardized framework for clinical practice.

**Methods:**

Under the leadership of Sichuan Cancer Hospital and in collaboration with national academic organizations, a multidisciplinary expert panel conducted a systematic literature review, nationwide surveys, and consensus meetings. The recommendations were formalized through a two-round Delphi process.

**Results:**

Three levels of pharmaceutical care are defined in this consensus, along with their corresponding implementation principles, including prioritization of high-risk patients, assignment of the highest applicable level when multiple criteria are met, and dynamic reassessment in response to changes in clinical status. The stratification system is established based on a comprehensive assessment across three core dimensions: 1) Pathophysiological conditions, including fertility preservation, age, body mass index, performance status, comorbid chronic diseases, concurrent infections, nutritional status and support, pain management, and hepatic or renal impairment; 2) Medication-related factors, encompassing antineoplastic agent toxicity risk and management, therapeutic drug monitoring, pharmacogenomics, clinically significant drug-drug interactions, polypharmacy, special administration routes or delivery devices, medication adherence, and complex medication issues; 3) Non-medication therapeutic interventions, such as radiotherapy, interventional therapy, surgery, and novel therapies (e.g., CAR-T therapy, tumor-infiltrating lymphocyte therapy). Specific recommendations for stratified pharmaceutical care were formulated based on these factors.

**Conclusions:**

This expert consensus establishes a standardized and practical framework for stratified pharmaceutical care in cancer patients, aiming to improve care quality, optimize resource allocation, and enhance patient outcomes in China. It may also serve as a reference model for international initiatives seeking to establish or refine standards for oncology pharmaceutical care.

## 1 Introduction

The global burden of cancer continues to rise, accompanied by a rapidly increasing demand for medical resources. China faces one of the highest cancer caseloads worldwide, with large numbers of newly diagnosed cases and cancer-related deaths, highlighting the urgent challenges and pressing needs in national cancer prevention and treatment.

Cancer management is a highly individualized and multidisciplinary process that involves multiple therapeutic modalities such as radiotherapy, chemotherapy, targeted therapy, and immunotherapy. In this context, pharmaceutical services play an increasingly pivotal role in optimizing treatment regimens, minimizing drug-related toxicities, and ensuring safe and effective medication use. Clinical pharmacists have therefore become indispensable contributors to improving therapeutic outcomes in oncology.

Pharmaceutical care, as the core component of pharmaceutical services, refers to direct, medication-related professional support provided by pharmacists to inpatients, aiming to enhance the safety, efficacy, and cost-effectiveness of drug therapy. In 2021, the National Health Commission of China issued the Standards for Pharmaceutical Care Services in Medical Institutions, which identified inpatients—particularly those with organ dysfunction, special physiological conditions, or malignancies—as the primary focus of pharmaceutical care. By implementing individualized and precision-oriented drug management, pharmaceutical care can effectively identify, prevent, and resolve drug-related problems, thereby reducing medication-related risks and improving clinical outcomes.

However, with the expanding population of cancer patients and limited clinical pharmacist resources—especially in developing regions—an urgent challenge arises: how to deliver high-quality pharmaceutical care efficiently and precisely. Stratified pharmaceutical care has emerged as a promising approach to address this issue. By establishing a standardized, evidence-based stratification framework—incorporating factors such as disease risk, medication complexity, and physiological status—patients can be assigned to different monitoring levels. This approach enables the accurate identification of high-risk patients, rational allocation of pharmacist resources, and enhanced workflow efficiency, ultimately improving care quality and overall service value.

Although stratified pharmaceutical care has been applied in selected populations such as respiratory diseases (Lian et al., 2024; Gong et al., 2023), perioperative patients (Jiang et al., 2023; Fang et al., 2022), and cardiovascular diseases (Wei et al., 2023), standardized guidelines for its implementation in oncology remain lacking. Existing recommendations based on hepatic/renal function, disease status, and medication profile (Bu et al., 2015; Xu et al., 2024) are insufficient to address the distinct complexity of cancer care, which involves highly individualized multi-drug regimens with cytotoxic, targeted, or immune-based agents; diverse and severe toxicity profiles; substantial interpatient heterogeneity; frequent comorbidities; and polypharmacy. Therefore, developing an evidence-based, oncology-specific stratified pharmaceutical care framework is both clinically necessary and practically urgent.

This expert consensus aims to fill this gap by integrating multidisciplinary expertise and clinical experience to propose a scientifically sound, pragmatic, and operational grading standard. Although this framework was developed to address the specific challenges and resource constraints within the Chinese healthcare system, the core principles of risk-based stratification are universal. This consensus therefore provides a foundational reference for standardizing and improving oncology pharmaceutical care in China, while also offering insights applicable to similar contexts globally.

## 2 Methods of consensus development

This consensus has been registered with the Practice Guideline REgistration for transPAREnCy (PREPARE, https://www.guidelines-registry.org/), under the registration number PREPARE-2024CN1097.

This consensus was initiated by Sichuan Cancer Hospital, in collaboration with the Chinese Society of Clinical Pharmacy, Chinese Medical Association; the Cancer Pharmacists Branch of Chinese Pharmacists Association; the Hospital Pharmacy Committee of Chinese Pharmaceutical Association; and the Oncology Clinical Pharmacy Committee of Sichuan Anti-Cancer Association. The multidisciplinary working group included oncology-specialized clinical pharmacists, medical oncologists, surgical oncologists, radiation oncologists, and experts in evidence-based medicine. The consensus development process included the following key components: a systematic evidence review and critical appraisal of existing pharmaceutical care grading frameworks; a nationwide practice survey collecting insights from frontline clinicians and pharmacists; structured consensus meetings to draft and refine recommendations; and a two-round Delphi consultation to achieve formal expert consensus. Detailed methodologies, including search strategies, survey instruments, participant demographics, and statistical analyses of consensus outcomes, are comprehensively documented in Supplementary Material 1.

Target Population: Pharmacists, physicians, nurses, and researchers engaged in the care of adult patients with confirmed solid tumors undergoing pharmacological or non-pharmacological antineoplastic therapy.

## 3 Basic principles of stratified pharmaceutical care

### 3.1 Definition of stratification levels

Building upon established practice of categorizing pharmaceutical care into three levels (Lian et al., 2024; Bu et al., 2015), this consensus introduces a novel, oncology-specific framework. This system provides a more tailored approach to address the clinical complexity of cancer patients through a comprehensive assessment of pathophysiological status, medication-related factors, and non-medication therapeutic interventions. The stratification, based on clinical priority and monitoring intensity, is defined as follows:

- Level 1 Pharmaceutical Care: Applies to patients with life-threatening or potentially disabling conditions where pharmacotherapy critically influences survival or other key clinical outcomes. This level mandates high-intensity, real-time monitoring and proactive intervention by a pharmacist.
- Level 2 Pharmaceutical Care: Indicated for severe or clinically significant conditions that are not immediately life-threatening but may significantly prolong hospitalization, increase costs, or compromise treatment adherence. It requires systematic evaluation, standardized monitoring, and active management of adverse events.
- Level 3 Pharmaceutical Care: Designed for patients in a relatively stable disease state but with identifiable medication-related risks. The objective is to enhance quality of life and disease control through periodic review, patient education, and adherence support.

### 3.2 Principles for stratified pharmaceutical care

Inclusion Threshold: Given the resource limitations in China, only patients classified as Level 3 or above are included in the pharmaceutical care scope.

Highest Applicable Level: When a patient meets the criteria for multiple levels, the highest applicable level of care is assigned.

Dynamic Reassessment: Any change in a patient’s treatment or clinical status should trigger an immediate reassessment of the care level to ensure its continued appropriateness.

## 4 Implementation of stratified pharmaceutical care

In accordance with the 2021 Pharmaceutical Care Service Standards for Medical Institutions (National Health Commission of China), and considering the distinct clinical characteristics, care requirements, and therapeutic complexity of oncology patients, as well as current service capacity and regulatory frameworks, we refined, prioritized, and operationalized the core implementation components for each stratification level (Table 1).

**TABLE 1:** Main items of stratified pharmaceutical care.

Pharmaceutical care items	Level 1 care	Level 2 care	Level 3 care
Pharmaceutical care frequency*	At least 3 times per week	At least 2 times per week	At least 1 time per week
Implementation items	Order review, medication reconciliation, medication education, adjustment of diagnosis and treatment plans, evaluation of the rationality of antineoplastic regimens, monitoring of therapeutic efficacy, adverse drug reactions, drug treatment processes, medication adherence, and individualized medication monitoring#	Order review, medication reconciliation, medication education, adjustment of diagnosis and treatment plans, evaluation of the rationality of antineoplastic regimens, monitoring of therapeutic efficacy, adverse drug reactions, drug treatment processes, and medication adherence	Order review, medication reconciliation, medication education, adjustment of diagnosis and treatment plans, and evaluation of the rationality of antineoplastic regimens

*: For patients with a hospitalization duration of less than 1 week, the frequency of pharmaceutical care shall follow the requirements for a 1-week cycle or be adjusted based on actual conditions. #: Individualized medication monitoring refers to therapeutic drug monitoring (TDM), drug metabolism-related gene testing, etc., with pharmaceutical care implemented based on test results.

## 5 Stratification criteria and details

### 5.1 Pathophysiological conditions

#### 5.1.1 Special physiological conditions

##### 5.1.1.1 Fertility preservation

Level 1 pharmaceutical care is recommended for cancer patients with fertility preservation needs scheduled for gonadotoxic therapy.

According to the International Federation of Gynecology and Obstetrics (FIGO), all forms of anticancer therapy may adversely affect fertility (Henry et al., 2023). Chemotherapy-induced gonadotoxicity primarily targets rapidly dividing germ cells, and the extent of gonadal damage depends on the treatment regimen, cumulative dose, and patient age (Elenkov and Giwercman, 2022). Although hormonal therapy itself lacks direct gonadotoxicity, declining ovarian reserve with advancing age and prolonged treatment duration may increase infertility risk. Therefore, patients should receive counseling regarding potential fertility risks and expected treatment duration before initiating endocrine therapy. Androgen deprivation therapy (ADT) causes hypogonadism and testosterone suppression, correlating with oligospermia/azoospermia and transient infertility (Colleoni et al., 2006; Fornier et al., 2005; Lambertini et al., 2019). Limited and heterogeneous data exist on targeted therapy-associated infertility risks. Certain agents—such as epidermal growth factor receptor tyrosine kinase inhibitors (EGFR-TKIs) and everolimus—may carry potential gonadotoxic risks, whereas no relevant fertility data have been reported for immune checkpoint inhibitors (Santaballa et al., 2022). The Spanish multidisciplinary consensus (Santaballa et al., 2022) summarized the fertility risks associated with specific anticancer treatment regimens, as presented in Supplementary Material 2.

##### 5.1.1.2 Advanced age

Level 3 pharmaceutical care is recommended for oncology patients aged ≥75 years.

In this consensus, individuals aged ≥65 years are defined as older adults, and those aged ≥75 years as very old adults, consistent with classifications used by the National Comprehensive Cancer Network (NCCN), World Health Organization (WHO), and relevant Chinese clinical guidelines. The *NCCN Guidelines for Older Adult Patients with Cancer (2023 Edition)* explicitly highlights that age-related changes in pharmacokinetics, pharmacodynamics, and organ function must be carefully considered when treating older cancer patients. Accordingly, Level 3 pharmaceutical care is recommended for cancer patients aged ≥75 years, who typically exhibit greater physiological vulnerability, polypharmacy, and higher risks of adverse drug reactions (ADRs).

##### 5.1.1.3 Body mass index (BMI)

Level 3 pharmaceutical care is recommended for cancer patients with underweight (BMI <18.5 kg/m^2^) or obesity (BMI ≥28 kg/m^2^).

BMI is a crucial factor influencing the efficacy and safety of antineoplastic agents, as extremes of body composition can lead to significant alterations in drug pharmacokinetics and toxicity risks. For patients with a high BMI, attention should be paid to reduced chemotherapeutic efficacy due to underdosing or increased toxicity from overdosing. Patients with a low BMI or cachexia require vigilance for drug accumulation toxicity caused by decreased clearance (Griggs et al., 2021). Pharmacists should develop individualized pharmaceutical care plans based on patients’ BMI onditions.

##### 5.1.1.4 Performance status

Level 3 pharmaceutical care is recommended for cancer patients with an Eastern Cooperative Oncology Group (ECOG) performance status ≥2 or Karnofsky Performance Status (KPS) ≤70.

Performance status is a key determinant of treatment tolerance and clinical prognosis in oncology patients. Individuals with impaired functional status (e.g., ECOG performance status ≥2) typically exhibit limited physical activity and require partial or full assistance in daily living. They are more susceptible to treatment-related toxicities, such as infection, fatigue, and myelosuppression, and frequently have comorbidities that complicate therapy. In contrast, patients with preserved functional capacity (e.g., ECOG performance status 0–1) generally maintain independence and tolerate therapy better, although ongoing monitoring is still needed. Therefore, during antineoplastic agents treatment decision - making and monitoring, patients’ performance status should be considered (Sehgal et al., 2021; Meyers et al., 2023).

#### 5.1.2 Special pathological conditions

##### 5.1.2.1 Comorbid chronic disease

Level 2 pharmaceutical care is recommended for cancer patients with unstable chronic comorbidities (e.g., hypertension, diabetes, chronic obstructive pulmonary disease [COPD]), whereas Level 3 care is recommended for those with relatively stable conditions.

Cancer patients with pre-existing chronic comorbidities often present significant therapeutic challenges in oncology care. For example, numerous anticancer therapies or agents may induce or exacerbate hypertension, and concurrent hypertension substantially elevates the risk of chemotherapy-induced cardiomyopathy and heart failure. Consequently, cardiovascular diseases have become one of the leading causes of morbidity and mortality among cancer survivors (Hypertension Committee of Chinese Research Hospital Association et al., 2024). For cancer patients with chronic comorbidities, if their underlying conditions are unstable before initiating anticancer therapy, or they require multiple concomitant medications for multiple chronic conditions. These factors can interact with anticancer therapies, thereby adversely impacting treatment outcomes. Thus, a stratified pharmaceutical care system should be implemented based on comprehensive clinical assessment of these patients with chronic comorbidities.

##### 5.1.2.2 Concurrent infection

For cancer patients with concurrent infections, stratified pharmaceutical care is recommended based on infection severity: Level 1 for severe infections, Level 2 for moderate infections, and Level 3 for mild infections.

For instance, in lung infections, their occurrence in patients with solid tumors is closely linked to tumor-specific characteristics and/or local or immunological defense impairments induced by anticancer therapies. Such associations lead to notable differences in preventive strategies, diagnostic methods, and therapeutic modalities between oncological and non-oncological populations. This results in distinct differences in prevention strategies, diagnostic approaches, and treatment modalities compared to non-oncological populations. The impact of lung infections varies significantly across different phases of anticancer treatment (pre-treatment, intra-treatment, and post-treatment phases), influencing patients’ subsequent treatment courses. Furthermore, the severity of infection dictates distinct clinical management strategies, and these strategies exert a profound influence on the development and adjustment of subsequent anticancer regimens. Therefore, implementing stratified pharmaceutical care based on infection severity in cancer patients is clinically warranted.

##### 5.1.2.3 Nutritional status and support

- Level 2 pharmaceutical care is recommended for cancer patients with severe malnutrition (Patient-Generated Subjective Global Assessment [PG-SGA] grade C), and Level 3 care is recommended for those with suspected or mild malnutrition (PG-SGA grade B).
- Level 3 pharmaceutical care is recommended for cancer patients with nutritional risk (Nutritional Risk Screening 2002 [NRS2002] score ≥3).
- Level 3 pharmaceutical care is recommended for cancer patients receiving parenteral nutrition or enteral tube-feeding.

Malnutrition in cancer patients significantly impacts clinical outcomes. A growing body of evidence confirms that patients with nutritional risk (NRS2002 score ≥ 3) derive significant benefits from nutritional interventions (Jie et al., 2010; Cong et al., 2015). The PG-SGA is a cancer patients-specific nutritional assessment tool. Its grading is strongly correlated with clinical thresholds for nutritional intervention, and PG-SGA C grade represents an absolute indication for nutritional therapy (Bauer et al., 2002). Nutritional intervention should follow the “Five-Step Therapy Model”, and patients receiving enteral tube feeding or parenteral nutrition (PN) require close monitoring, as key parameters of enteral nutrition (EN) formulas—including type, infusion rate, temperature, and osmolarity—directly affect patients’ tolerance and treatment adherence (Chinese Society for Oncological Nutrition and Supportive Care et al., 2023). Furthermore, concomitant use of other oral medications during tube feeding further increases the complexity of EN therapy. PN formulations are high-alert medications characterized by high concentration and hyperosmolarity, typically administered intravenously as total nutrient admixtures (TNAs). Inappropriate PN prescriptions may lead to suboptimal therapeutic efficacy or severe metabolic complications such as hyperglycemia and fat overload syndrome. Excessive electrolyte concentrations can destabilize lipid emulsions, while compounding errors may generate insoluble precipitates (e.g., calcium phosphate)—a life-threatening hazard if infused (Chen, 2024). Therefore, the appropriate level of pharmaceutical care for cancer patients should be comprehensively determined based on nutritional risk, severity of malnutrition, and type of nutritional support.

##### 5.1.2.4 Pain management

Level 2 pharmaceutical care is recommended for cancer patients, who are opioid - naive, with opioid - related safety risk factors (e.g., renal/hepatic dysfunction, respiratory impairment, sleep apnea, poor performance status), or with inadequate pain control despite opioid use.

In the pharmaceutical care grading for cancer pain management, three key factors must be considered: pain severity, type of analgesic agents, and the patient’s baseline clinical status. Pain severity assessment—typically via the Numerical Rating Scale (NRS) or Visual Analog Scale (VAS)—is fundamental as it quantifies pain and informs analgesic plans (Pain Physician Branch o f Chinese Medical Doctor Association et al., 2023). Additional critical considerations include the pharmacokinetic properties of analgesics and their inherent safety profiles. Non - opioid drugs usually have fewer adverse effects, while opioids carry a certain risk (Qin et al., 2019). The patient’s physiological state significantly impacts the absorption, distribution, metabolism, and excretion of opioids; impaired drug metabolism can lead to drug accumulation, which may exacerbate underlying comorbidities. Thus, stratifying pharmaceutical care based on pain severity, analgesic agents, and baseline status effectively prevents and manages ADRs, thereby ensuring medication safety and improving the patient’s quality of life.

##### 5.1.2.5 Hepatic/renal impairment

Stratified pharmaceutical care is recommended for cancer patients with hepatic or renal impairment: Level 1 for severe impairment, Level 2 for moderate impairment, and Level 3 for mild impairment.

Hepatic or renal dysfunction significantly compromises both the efficacy and safety of antineoplastic agents by impairing their metabolism and excretion. The severity of hepatic and renal impairment is positively correlated with the risk of serious adverse events; moreover, inadequate drug clearance in severe dysfunction may lead to suboptimal therapeutic outcomes due to altered drug exposure. Therefore, the appropriate level of pharmaceutical care should be determined based on the patient’s hepatic and renal function status (Chinese Society of Clinical Pharmacy, 2024).

Clinical Stratification of Liver and Renal Impairment:

Severe:

⁃ *Renal:* Creatinine clearance (CLcr) ≤30 mL·min^-1^
⁃ *Hepatic:* Alanine transaminase (ALT)/aspartate transaminase (AST)/alkaline phosphatase (ALP) > 5 × upper limit of normal (ULN), or total bilirubin (TBIL) > 3 × ULN, or Child-Pugh C grade

Moderate:

⁃ *Renal:* 30 mL·min^-1^ < CLcr ≤60 mL·min^-1^
⁃ *Hepatic:* ALT/AST/ALP > 2–4×ULN or TBIL > 2–3×ULN or Child-Pugh B grade

Mild:

⁃ *Renal:* 60 mL·min^-1^ < CLcr ≤90 mL·min^-1^
⁃ *Hepatic:* ALT/AST/ALP > 1–2×ULN or TBIL > 1–2×ULN or Child-Pugh A grade

### 5.2 Medication-related factors

#### 5.2.1 Antineoplastic agents toxicity risk management

Stratified pharmaceutical care is recommended for cancer patients receiving potentially toxic antineoplastic agents, taking into account: (a) the agent’s toxicity profile (spectrum, incidence, severity, and reversibility); (b) patient-specific characteristics (comorbidities, age, and prior radiotherapy); and (c) complexity of clinical management (monitoring, prophylaxis, regimen adjustments, and prognosis).

##### 5.2.1.1 Cardiovascular system

Cardiovascular toxicity encompasses a wide spectrum of manifestations, including heart failure, myocardial injury, arrhythmias, hypertension, and thrombosis. Based on the cardiotoxic potential of antineoplastic agents, and consideration of modifiable or non-modifiable exacerbating risk factors, stratified care is warranted. Cardiovascular toxicity risk factors identified in Chinese and international guidelines are largely consistent (Armenian et al., 2017; Curigliano et al., 2020; Chinese Society of Clinical Oncology Guidelines Working Committee, 2021; Chinese Society of Clinical Oncology Guidelines Working Committee, 2023): a multidimensional assessment—encompassing combination antitumor therapy, pre-existing cardiovascular disease, and advanced age—is required. Overall, the cardiovascular toxicity risk factors of antineoplastic agents include, but are not limited to, the following: history of impaired cardiac function/heart disease, advanced age, concurrent radiotherapy involving the heart, cardiac tumors, and the presence of two or more uncontrolled cardiovascular risk factors (hypertension, diabetes, dyslipidemia, obesity, etc.). For risk factors of cardiovascular toxicity other than cardiac toxicity caused by antineoplastic agents, the European Society of Cardiology guideline can be consulted (Lyon et al., 2022).

Level 2 pharmaceutical care is recommended if any of the following criteria are met:

- Use of anthracyclines (e.g., doxorubicin, epirubicin) with high-risk cardiac toxicity, in the presence of cardiac toxicity risk factors.
- Use of antineoplastic agents with moderate-risk cardiac toxicity (e.g., trastuzumab, fluorouracil), in patients with two or more cardiac toxicity risk factors.
- Patients presenting with symptoms such as myasthenia and myositis after the use of immune checkpoint inhibitors, who are suspected of developing immune-mediated myocarditis.

Level 3 pharmaceutical care is recommended if Level 2 criteria are not met but any of the following criteria are fulfilled:

- Patients receiving high cumulative doses of anthracyclines (doxorubicin ≥250 mg/m^2^, epirubicin ≥600 mg/m^2^).
- Sequential administration of anthracyclines followed by trastuzumab.
- Use of antineoplastic agents with potential cardiotoxicity (excluding anthracyclines) in patients with cardiotoxicity risk factors.
- Use of immune checkpoint inhibitors (ICIs, characterized by low incidence but high mortality of cardiotoxicity) in patients with additional risk factors (e.g., combination immunotherapy with programmed cell death protein 1 (PD-1) inhibitor plus cytotoxic T-lymphocyte-associated protein 4 (CTLA-4) inhibitor, thymic tumors).
- Use of antineoplastic agents with minimal cardiotoxicity but high incidence of other cardiovascular toxicities (e.g., hypertension caused by anti-angiogenic agents such as bevacizumab), in patients with risk factors that may exacerbate cardiovascular toxicity.

##### 5.2.1.2 Respiratory system

Respiratory toxicities associated with antineoplastic agents primarily encompass interstitial lung disease, pulmonary fibrosis, organizing pneumonia, non-cardiogenic pulmonary edema, diffuse alveolar hemorrhage, and immune-related pneumonia (Spagnolo et al., 2022; Ma and Dai, 2022; Shannon et al., 2020). High-risk factors for antineoplastic agent-induced respiratory toxicity include, but are not limited to: age >60 years, baseline pulmonary lesions, prior pulmonary surgery history, impaired respiratory function, prior pulmonary radiation exposure, high fraction of inspired oxygen (FiO_2_), and renal insufficiency. Risk stratification of respiratory toxicity requires comprehensive assessment of factors including antineoplastic drug type, cumulative dose, treatment course, combination therapy, history of lung disease, and respiratory function (Schwaiblmair et al., 2012; Se et al., 2016; Hasegawa et al., 2016; Nakamura et al., 2009).

Level 2 pharmaceutical care is recommended if any of the following criteria are met:

- Cumulative exposure dose of bleomycin ≥400 IU.
- Cumulative exposure dose of carmustine ≥1,500 mg/m^2^.
- Cumulative exposure dose of mitomycin ≥50 mg/m^2^.

Level 2 pharmaceutical care is recommended if any high-risk factor is present along with any of the following criteria; if no high-risk factors are present, Level 3 pharmaceutical care is recommended:

- Cumulative exposure dose of bleomycin <400 IU.
- Cumulative exposure dose of carmustine <1,500 mg/m^2^.
- Cumulative exposure dose of mitomycin <50 mg/m^2^.
- Radiotherapy (with a focus on thoracic irradiation) combined with chemotherapy (primarily comprising anthracyclines, paclitaxel, gemcitabine, and pemetrexed).

Level 3 pharmaceutical care is recommended if any of the following criteria are met:

- Treatment with PD-1/ programmed cell death ligand 1 (PD-L1) inhibitors, CTLA-4 inhibitors, or *Bacillus* Calmette-Guérin (BCG).
- Treatment with EGFR-TKIs, anaplastic lymphoma kinase (ALK) inhibitors, ROS proto-oncogene 1 (ROS1) inhibitors, neurotrophic tyrosine receptor kinase (NTRK) inhibitors, rearranged during transfection (RET) inhibitors, mesenchymal-epithelial transition factor (MET) inhibitors, breakpoint cluster region-Abelson (BCR-ABL) inhibitors, mammalian target of rapamycin (mTOR) inhibitors, or antibody-drug conjugates (ADCs).
- Targeted therapy with trametinib, rituximab, bevacizumab, pazopanib, duvelisib, avapritinib, gilteritinib, or amivantamab.
- Chemotherapy with cyclophosphamide, lomustine, gemcitabine, or melphalan.

##### 5.2.1.3 Digestive system

Digestive system toxicities associated with antineoplastic agents are primarily classified into gastrointestinal toxicities (e.g., nausea/vomiting, diarrhea, oral mucositis) and hepatobiliary toxicities (e.g., drug-induced liver injury). The care level should be determined based on severity of adverse reaction risk, patient-specific factors (e.g., comorbidities, genetic predisposition) and concurrent medications or other therapies (Zhang, 2024)^.^

Level 3 pharmaceutical care is recommended if any of the following criteria are met:

- Use of antineoplastic agents with a high risk of gastrointestinal toxicity (i.e., a high incidence of gastrointestinal toxicity), along with other high-risk factors (e.g., brain metastases, intestinal obstruction, anxiety disorders, a history of severe vomiting following prior chemotherapy, and irritable bowel syndrome).
- Use of antineoplastic agents with high hepatotoxic risk, in patients with underlying liver disease and/or concurrent use of other agents with significant hepatotoxic potential (e.g., anti-tuberculosis agents, statins).

##### 5.2.1.4 Endocrine system

Endocrine toxicities induced by antineoplastic agents primarily include thyroiditis, hypophysitis, metabolic disorders, and perimenopausal symptoms (National Comprehensive Cancer Network (NCCN), 2024a). Risk stratification of endocrine toxicities requires comprehensive requires comprehensive assessment of factors including antineoplastic agent type, dosage, treatment duration, combination therapy, and pre-existing endocrine disorders.

Level 2 pharmaceutical care is recommended if any of the following criteria are met:

- Use of antineoplastic agents with a high incidence of endocrine toxicities, combined with one or more high-risk factors for such toxicities (e.g., poorly controlled pre-existing endocrine disorders; radiotherapy to endocrine glands, or a history of endocrine-related adverse events).

Level 3 pharmaceutical care is recommended if Level 2 criteria are not met but any of the following criteria are fulfilled:

- Use of ICIs.
- Use of pan-targeted TKIs with vascular endothelial growth factor receptor (VEGFR) inhibitory activity.
- Use of ALK inhibitors.
- Use of phosphatidylinositol 3-kinase (PI3K), protein kinase B (AKT), or mechanistic target of rapamycin (mTOR) inhibitors.
- Use of bone-modifying agents (e.g., bisphosphonates or denosumab) for ≥2 years.
- Use of endocrine therapy for cancer f9or ≥5 years.
- Use of VEGFR2 monoclonal antibody (ramucirumab).

##### 5.2.1.5 Hematological system

Hematotoxicity caused by antineoplastic agents is one of the most common ADRs in clinical practice, characterized by typical clinical manifestations such as leukopenia, thrombocytopenia, and anemia. For antineoplastic agent-related hematotoxicity, a comprehensive assessment should be conducted considering factors such as the risk and severity of hematological toxicity, concomitant medications, treatment methods, and patient characteristics (e.g., prior history of hematotoxicity). This consensus summarizes and refines recommendations from relevant guidelines to systematically evaluate the hematotoxic potential of antineoplastic drugs.

Level 2 pharmaceutical care is recommended if any of the following criteria are met:

- A history of neutropenia during previous cancer treatment, with the current use of high-risk drugs or regimens for neutropenia induction (e.g., MVAC regimen [methotrexate + vinblastine + doxorubicin + cisplatin], FOLFOXIRI regimen [fluorouracil + leucovorin + oxaliplatin + irinotecan], TAC regimen [docetaxel + doxorubicin + cyclophosphamide], and other similar regimens).
- A history of Grade 3/4 chemotherapy-induced thrombocytopenia (CTIT) during previous cancer treatment, and the current use of drugs or regimens with an incidence of Grade 3/4 CTIT ≥30% (e.g., niraparib, bortezomib, topotecan + cisplatin, and other analogous agents/regimens).
- A history of severe to life-threatening anemia during prior cancer treatment, with current use of agents such as poly (ADP-ribose) polymerase (PARP) inhibitors, nivolumab, and others.

Level 3 pharmaceutical care is recommended if Level 2 criteria are not met but any of the following criteria are fulfilled:

- A history of neutropenia during prior cancer treatment, with current use of intermedium-risk drugs or regimens for neutropenia induction (e.g., AC-T regimen [doxorubicin + cyclophosphamide followed by paclitaxel], docetaxel, irinotecan, and other similar agents/regimens).
- A history of Grade 3/4 CTIT during prior cancer treatment, with current use of drugs or regimens with an incidence of Grade 3/4 CTIT of 11%–30% (e.g., trastuzumab emtansine, gemcitabine, temozolomide, and other analogous agents/regimens).
- Use of high-risk drugs or regimens for neutropenia.
- Use of drugs or regimens with an incidence of Grade 3/4 CTIT ≥30%
- Concurrent use of medium-risk drugs or regimens for neutropenia and drugs or regimens with an incidence of Grade 3/4 CTIT of 11%–30%
- A history of mild or moderate anemia during previous cancer treatment, with current receipt of agents such as ADCs, platinum-containing agents, PARP inhibitors, immune checkpoint inhibitors, and others.

##### 5.2.1.6 Immune system

Certain antineoplastic agents modulate the activation and migration of tumor cells by regulating the biological activities of cytokines or cell receptors in the immune system, thereby modulating interactions between immune cells (Naimi et al., 2022; Shin et al., 2023; Polak et al., 2024). However, such antineoplastic drugs interfere with the normal activities of these molecules, thus disrupting the host’s immune response, which not only triggers autoimmune diseases (including cutaneous, gastrointestinal, hepatic, endocrine events, and other uncommon inflammatory events) (Chinese Society of Oncology and Chinese Medical Association, 2023) but also increases the risks of bacterial, fungal, and viral infections (Hematopoietic Stem Cell Application Group et al., 2024; Chinese Society of Clinical Oncology, 2024). When stratifying pharmaceutical care for antineoplastic agent-induced immune system toxicities, agent-specific factors (e.g., dosage, combination or sequential administration of immunotherapy with other treatments, and treatment duration) and patient-specific factors (e.g., age, pre-existing autoimmune diseases, and concurrent infections [e.g., hepatitis B virus (HBV), hepatitis C virus (HCV), tuberculosis, Pneumocystis jirovecii pneumonia]) should be comprehensively evaluated.

Level 1 pharmaceutical care is recommended if any of the following criteria are met:

- Patients undergoing hematopoietic stem cell transplantation.
- Patients undergoing chimeric antigen receptor T-cell (CAR-T) therapy.

Level 2 pharmaceutical care is recommended if any of the following criteria are met:

- Patients with active HBV infection (HBsAg-positive) or inactive HBV infection (HBcAg-positive and HBsAg-negative) receiving anti-B cell agents (e.g., rituximab) are at increased risk of HBV reactivation.

Level 3 pharmaceutical care is recommended if Level 2 criteria are not met but any of the following criteria are fulfilled:

- Patients with hematological malignancies using anti-B cell drugs, who are at increased risk of infections (e.g., Pneumocystis pneumonia, cryptococcal infection, Epstein-Barr virus [EBV] infection, and cytomegalovirus [CMV] infection, etc.).
- Patients with pre-existing autoimmune diseases receiving ICIs.

##### 5.2.1.7 Urinary system

Antineoplastic agents can induce nephropathy and electrolyte disturbances by damaging the glomeruli, renal tubules, or renal interstitium. Clinical manifestations range from asymptomatic serum creatinine elevation to acute kidney injury requiring dialysis (Yanagita et al., 2024). Assessment of nephrotoxicity risk requires comprehensive evaluation of agent-specific characteristics (e.g., type, dose), treatment modalities (e.g., combination therapy, radiotherapy), and patient-related factors (e.g., intravascular volume depletion, urinary tract obstruction, pre-existing renal disease).

Level 2 pharmaceutical care is recommended if any of the following criteria are met:

- Use of high-risk or high-dose nephrotoxic antineoplastic agents (e.g., ifosfamide, cisplatin, high-dose cyclophosphamide [single dose ≥1 g/m^2^ or cumulative dose ≥10 g/m^2^], high-dose methotrexate [1–15 g/m^2^]) (Sun et al., 2021; Crona et al., 2017; Widemann and Adamson, 2006).
- Use of potentially nephrotoxic systemic antineoplastic in combination with any of the following risk factors: Intravascular volume depletion (e.g., fluid loss, edema); Concurrent nephrotoxic medications (e.g., aminoglycosides, nonsteroidal anti-inflammatory drugs [NSAIDs], proton pump inhibitors [PPIs]); Urinary tract obstruction; Pre-existing or acquired renal disease.
- Use of high-risk nephrotoxic antineoplastic agents combined with radiotherapy targeting the urinary system.

Level 3 pharmaceutical care is recommended if Level 2 criteria are not met but any of the following criteria are fulfilled:

- A history of urinary system impairment (e.g., chronic kidney disease, bladder dysfunction) receiving potentially nephrotoxic antineoplastic agents.
- Prior exposure to potentially nephrotoxic antineoplastic agents, with current switching to another agent within the same nephrotoxic class.
- Use of potentially nephrotoxic antineoplastic agents (e.g., carboplatin, non-high-dose methotrexate, non-high-dose cyclophosphamide, oxaliplatin, gemcitabine, capecitabine, pemetrexed, irinotecan, bevacizumab, abemaciclib, lenvatinib) combined with radiotherapy targeting the urinary system.
- Development of subclinical renal impairment during treatment (e.g., microalbuminuria, elevated serum creatinine) with ongoing therapy required (Yanagita et al., 2024).

##### 5.2.1.8 Nervous system

Antineoplastic agents can induce neurotoxicity via direct or indirect mechanisms, significantly impacting the quality of life of cancer patients (Lyu et al., 2024; Zhao et al., 2015). Evaluation of the severity of neurotoxic adverse events requires comprehensive assessment of multiple factors, including patient-specific characteristics, antineoplastic agent dosage, and treatment regimens.

Level 2 pharmaceutical care is recommended if any of the following criteria are met:

- Use of high-risk neurotoxic antineoplastic agents administered at cumulative dose or treatment durations associated with neurotoxicity risk:

(a) Cisplatin >350 mg/m^2^ cumulative dose.
(b) Oxaliplatin 780–850 mg/m^2^ cumulative dose.
(c) Paclitaxel 250–300 mg/m^2^ cumulative dose.
(d) Docetaxel >100 mg/m^2^ cumulative dose.
(e) Vincristine 2–6 mg/m^2^ cumulative dose.
(f) Bortezomib 16–26 mg/m^2^ cumulative dose.

- Use of high-risk neurotoxic drugs in combination with any of the following high-risk neurological factors:

(a) Recurrent Grade 1–2 neurotoxicity.
(b) History of Grade ≥3 neurotoxicity.
(c) Pre-existing neurological disorders.
(d) Impaired liver/kidney function.

Level 3 pharmaceutical care is recommended if Level 2 criteria are not met but any of the following criteria are fulfilled:

- Chemotherapy drugs with a >1% incidence of neurotoxicity.
- ADCs containing monomethyl auristatin E (MMAE) as the cytotoxic payload.
- Third-generation ALK TKIs.
- PARP inhibitors.
- Immune checkpoint inhibitors (Guidon et al., 2021).
- Adoptive cell therapy (ACT).

##### 5.2.1.9 Skin and mucosal system

Antineoplastic agents can induce skin and mucosal toxicities, including rash, pruritus, paronychia, hand-foot syndrome, hand-foot skin reaction, psoriasis-like eruption, vitiligo, and other manifestations. With the exception of amivantamab—a drug associated with frequent skin reactions and complex management—the skin and mucosal toxicity risk of most antineoplastic agents is manageable (Lacouture et al., 2021; Dermatology and Venereology Branch of Shanghai Medical Association and Tumor Target Molecule Branch of Shanghai Medical Association, 2023; Dermatology and Venereology Branch of Chinese Medical Association and Chinese Dermatologist AssociationChinese Dermatologist Association of the Chinese Medical Doctor Association Dermatology Division of the Chinese Geriatrics Society, 2024). However, patients with high-risk factors are at increased risk and require close monitoring. Comprehensive evaluation of risk factors for antineoplastic agent-induced skin and mucosal toxicities is required (Lacouture et al., 2021; Dermatology and Venereology Branch of Shanghai Medical Association and Tumor Target Molecule Branch of Shanghai Medical Association, 2023; Dermatology and Venereology Branch of Chinese Medical Association and Chinese Dermatologist Association Chinese Dermatologist Association of the Chinese Medical Doctor Association Dermatology Division of the Chinese Geriatrics Society, 2024), including treatment-related factors (e.g., polypharmacy, concurrent radiotherapy) and pre-existing skin conditions. The risk is The risk is elevated when agents with overlapping skin toxicity profiles are combined; for example, the combination of capecitabine and anlotinib increases the risk of hand-foot skin toxicity. Concurrent radiotherapy with paclitaxel, docetaxel, anthracyclines, fluorouracil, or EGFR inhibitors elevates the incidence of radiation dermatitis. Treatment with immune checkpoint inhibitors increases the risk of skin reaction development or relapse in patients with autoimmune skin diseases.

Level 2 pharmaceutical care is recommended if any of the following criteria are met:

- Patients receiving antineoplastic agents with a high propensity for skin toxicities and complex management (e.g., amivantamab).
- Patients with a history of Grade 3/4 mucocutaneous toxicity following prior antineoplastic therapy who require re-challenge with the same or analogous agents.
- Combination of agents with overlapping skin toxicity profiles (e.g., capecitabine plus anlotinib, which increases the risk of hand-foot skin toxicity).

Level 3 pharmaceutical care is recommended if any of the following criteria are met:

- Patients receiving antineoplastic agents with a higher risk of skin toxicity (e.g., capecitabine, regorafenib, everolimus) in the presence of exacerbating risk factors; or concurrent radiotherapy with paclitaxel, docetaxel, anthracyclines, fluorouracil, or EGFR inhibitors.
- Patients with pre-existing autoimmune skin conditions who are at increased risk of developing or experiencing a relapse of skin reactions when treated with ICIs.

##### 5.2.1.10 Others

For antineoplastic agents on the market for less than 5 years or those in active clinical trials at our institution, there is limited post-marketing surveillance and clinical experience. In accordance with the hierarchical management of such agents, we therefore implement a stratified pharmaceutical care model to mitigate the unique risks they pose. These risks arise from both their inherent toxicities and the current lack of comprehensive clinical data.

Level 2 pharmaceutical care is recommended if any of the following criteria are met:

- New antineoplastic agents with severe ADRs (Grade 3–4) with an incidence >30%, as specified in the prescribing information or randomized controlled trials.
- New anti-tumor drugs with warnings in the prescribing information related to ADRs.
- Agents with severe but rare ADRs reported in the drug instructions or literature.
- Agents for the first use in the medical institution.
- Agents with frequent off-label use.
- Agents with a broad target spectrum.

#### 5.2.2 Antineoplastic agents toxicity management

For cancer patients with adverse events (AEs) attributed to antineoplastic agents, stratified pharmaceutical care for drug-related toxicities is categorized into three levels in accordance with the Common Terminology Criteria for Adverse Events version 5.0 (CTCAE v5.0):

- Level 1 pharmaceutical care is implemented for life-threatening or disabling toxicities that necessitate permanent discontinuation of antineoplastic therapy.
- Level 2 pharmaceutical care is implemented for patients experiencing severe or clinically significant toxicities that are not immediately life-threatening but may result in prolonged hospitalization, increased treatment costs, or reduced adherence. These toxicities often necessitate temporary interruption or dose adjustment of anticancer therapy, with treatment resumption allowed only after a comprehensive benefit–risk assessment.
- Level 3 pharmaceutical care is implemented for all other antineoplastic-related toxicities that do not meet the criteria for Level 1 or Level 2.

Antineoplastic agent-induced toxicity is a key factor influencing medication safety and patient quality of life. The NCI CTCAE, as an internationally recognized toxicity assessment system, provides an objective and standardized framework for toxicity management.

Special vigilance is required for life-threatening or disabling toxicities, including but not limited to: CTCAE Grade ≥2 immune-related adverse events (irAEs; e.g., myocarditis, Guillain-Barré syndrome, transverse myelitis, encephalitis, hemolytic anemia), CTCAE Grade 3 toxicities (e.g., pneumonitis, acute pancreatitis, bullous dermatologic toxicity, myasthenia gravis, severe hypersensitivity reactions) and CTCAE Grade 4 non-hematologic toxicities. These toxicities significantly impact patient survival or treatment outcomes due to the time-toxicity relationship and thus require proactive pharmacist intervention, warranting Level 1 (highest-level) pharmaceutical care. For hematologic toxicities such as Grade 3/4 neutropenia (absolute neutrophil count [ANC] <1 × 10^9^/L) or thrombocytopenia (<50 × 10^9^/L), which are not immediately life-threatening but may result in serious infections or bleeding, prolonged hospitalization, increased healthcare costs, and reduced treatment adherence, active pharmacist involvement is essential to optimize therapeutic outcomes. Such cases require Level 2 (intermediate-level) pharmaceutical care. Implementing CTCAE-based stratified pharmaceutical care improves the timeliness and accuracy of the antineoplastic toxicity identification and intervention, refines antineoplastic agent management strategies, and further ensures the continuity of antineoplastic therapy and patient safety during treatment.

#### 5.2.3 Therapeutic drug monitoring (TDM) and pharmacogenomics

- Level 1 pharmaceutical care is recommended for cancer patients with drug concentrations outside the target range and exhibiting toxicity.
- Level 2 pharmaceutical care is recommended for cancer patients with concentrations within the target range but exhibiting toxicity, or outside the range without toxicity.
- Level 3 pharmaceutical care is recommended for cancer patients with concentrations within the target range and without toxicity.
- Level 2 pharmaceutical care is recommended for cancer patients with abnormal drug metabolism genotypes (ultra-rapid or poor metabolizers).

Therapeutic drug monitoring (TDM) is indicated for antineoplastic agents with a narrow therapeutic window, significant interindividual metabolic variability, multiple drug interactions, and a clear dose-effect relationship, such as methotrexate, fluorouracil, paclitaxel, docetaxel, imatinib, sunitinib, lenvatinib, and everolimus, and other agents (Groenland et al., 2021). However, the clinical applicability of TDM varies among these drugs. For instance, TDM for methotrexate is well-standardized and routinely available, whereas for tyrosine kinase inhibitors (e.g., imatinib, sunitinib) it relies on more complex technologies (e.g., LC-MS/MS) and is typically accessible in specialized centers. In contrast, TDM for taxanes (e.g., paclitaxel) remains primarily a research tool due to technical challenges. TDM results are pivotal for guiding the optimization of therapeutic regimens. Drug concentrations within the target range indicates effective treatment with minimal a risk of toxicity. Conversely, concentrations outside the target range suggests insufficient or excessive dosing, or the presence of other factors influencing drug levels, thereby increasing the risk of treatment failure or toxicity. Polymorphisms in drug-metabolizing enzymes, primarily those of the CYP450 family, as well as in drug transporters such as ATP-binding cassette proteins, significantly influence the pharmacokinetics of antineoplastic agents. Dosage adjustments or even drug substitution based on the function of different genotypes of metabolic enzymes are necessary. The Clinical Pharmacogenetics Implementation Consortium (CPIC) has also issued a series of guidelines to support genotype-directed drug therapy. Integrating TDM results, treatment response, and drug metabolism genotypes into the stratification of pharmaceutical care can enhance the precision of tumor treatment, reduce drug-related toxic risks, and optimize healthcare resource utilization.

#### 5.2.4 Clinically significant drug-drug interactions (DDIs)

Level 2 pharmaceutical care is recommended for cancer patients with clinically significant drug–drug interactions (DDIs) that cannot be avoided, whereas Level 3 care is recommended when interactions are mitigated but still require monitoring.

Cancer patients—particularly older adults—often receive multiple concomitant medications, and many antineoplastic agents with narrow therapeutic indices are highly susceptible to DDIs. Such interactions may result in decreased therapeutic efficacy or increased toxicity. To address these challenges, a Dutch multidisciplinary expert panel published the Evidence- and Consensus-Based Guideline for Drug-Drug Interactions with Antineoplastic Agents in 2022 (van Leeuwen et al., 2022). Through systematic literature review, the guideline identified 290 potential DDIs and provided categorized management recommendations based on current evidence. This guideline serves as a valuable reference for pharmacists conducting medication reviews during pharmaceutical care of cancer patients.

#### 5.2.5 Polypharmacy

Level 2 pharmaceutical care is recommended for cancer patients prescribed ≥10 non-antineoplastic medications, whereas Level 3 care is recommended for those prescribed 5–9 antineoplastic medications.

Polypharmacy is typically defined as the concurrent use of five or more medications, while hyperpolypharmacy refers to ten or more (Shen et al., 2024). A Danish cohort study demonstrated that multimorbidity and polypharmacy are associated with increased mortality among certain cancer patient populations (Thomsen et al., 2023). Current evidence indicates that integrating pharmacists into patient-centered healthcare models represents an effective strategy for managing polypharmacy. Furthermore, studies have shown that pharmacist-led polypharmacy management and deprescribing interventions in geriatric oncology clinics resulted in symptom improvement in two-thirds of patients and achieved significant potential cost savings per patient (Thomsen et al., 2023). Therefore, stratified pharmaceutical care should be implemented for cancer patients with polypharmacy.

#### 5.2.6 Special routes of administration or delivery devices

Level 2 pharmaceutical care is recommended for cancer patients receiving enteral tube feeding with enteric-coated preparations, sustained-/controlled-release formulations, pharmaceutically incompatible medications, hyperosmolar fluids (>500 mOsm/L), or drugs prone to precipitation, as well as for those with gastrointestinal dysfunction requiring enteral feeding.

Enteral administration via feeding tubes is associated with significant risks, including drug-nutrient physicochemical interactions, disruption of dosage form integrity, and tube occlusion, which may substantially compromise efficacy or increase toxicity. Consequently, intensified pharmaceutical care is essential. Medication regimens must be adjusted based on the specific characteristics of the nasogastric or gastric tube (Blumenstein et al., 2014).

Liquid formulations (e.g., solutions or suspensions) are preferred for their ease of tube administration and predictable absorption. If solid dosage forms are used, only those that can be safely crushed or are designed for dispersion should be considered, with rigorous assessment of post-manipulation stability and potential bioavailability changes (Klang, 2023).

Critical assessment of tube characteristics (e.g.,,material, internal diameter, port location) relative to drug properties is imperative during enteral administration to prevent occlusion or maldistribution (Klang, 2023; Bandy et al., 2019) High-risk medications require intensified care, including: enteric-coated preparations,sustained-/controlled-release formulations, hyperosmolar agents, drugs with known incompatibilities with enteral nutrition formulas. Administration of these agents typically requires interval dosing or regimen modification. Pharmacists should lead multidisciplinary collaboration to establish standardized protocols (e.g., flushing procedures, drug administration sequences, monitoring plans).

#### 5.2.7 Medication adherence

Level 3 pharmaceutical care is recommended for cancer patients with suboptimal medication adherence (e.g., missed doses, incorrect administration, self-adjusted dosing, or treatment refusal).

Medication adherence among cancer patients directly impacts the efficacy and safety of antineoplastic therapy. Pharmacists should improve medication adherence through medication education, regimen simplification, and regular follow-up while monitoring therapeutic outcomes. Key pharmacist-led interventions include chemotherapy counseling, risk assessment and patient education, monitoring of ADRs and drug-drug interactions (DDIs), adherence evaluation. Pharmacist-led interventions significantly reduce medication-related problems (MRPs) in patients receiving antineoplastic therapy (Fentie et al., 2024). Pharmacists play a vital role in multidisciplinary oncology care by improving quality of life, reducing the risk of drug interaction, enhancing medication adherence rates, and increasing patient’s medication-related knowledge (Bandiera et al., 2024). Standardized tools (e.g., the 8-item Morisky Medication Adherence Scale [MMAS-8]) are recommended for adherence assessment, combined with personalized interventions based on patients’ cognitive levels and social support systems (Author anonymous, 2023).

#### 5.2.8 Complex medication issues

Level 2 pharmaceutical care is recommended for cancer patients with complex medication issues requiring collaborative decision-making by two or more clinical pharmacists from different specialties (e.g., oncology, anti-infectives, enteral/parenteral nutrition).

These patients require multidisciplinary team (MDT)-based individualized therapy plan, with dynamic assessment of drug interactions, cumulative toxicity risks, and treatment efficacy. The NCCN guidelines emphasize MDT collaboration—including palliative care physicians, nurses, dietitians, social workers, mental health professionals, and pharmacists—in managing complex medication issues in oncology (National Comprehensive Cancer Network (NCCN), 2024b; National Comprehensive Cancer Network (NCCN), 2025; National Comprehensive Cancer Network (NCCN), 2024c). Clinical pharmacists from diverse specialty areas play an essential role in identifying and managing drug interactions, mitigating toxicities, and implementing individualized dose adjustments. Their expertise ensures optimized therapeutic outcomes and enhanced patient safety across all phases of cancer treatment.

### 5.3 Non-medication therapeutic interventions

#### 5.3.1 Conventional non-medication interventions

Level 1 pharmaceutical care is recommended for cancer patients experiencing life-threatening or disabling complications or AEs from non-medication treatments (e.g., radiotherapy, interventional therapy, or surgery); Level 2 care is recommended for severe events but non-life-threatening complications or AEs; and Level 3 care is recommended for mild-to-moderate complications or AEs. (*When complications such as infection or hepatic/renal insufficiency occur, stratified care should be determined according to the corresponding criteria*.)

#### 5.3.2 Novel non-medication interventions

Level 2 pharmaceutical care is recommended for cancer patients receiving immunocellular therapies (e.g., CAR-T, tumor-infiltrating lymphocyte [TIL]), with Level 1 care recommended for associated complications. For patients receiving gene therapy, targeted radionuclide therapy (e.g., ^177^Lu-PSMA), or physical ablation/photodynamic therapy, Level 1 care is recommended for severe complications, and Level 2 care for mild-to-moderate complications.

Given the diverse modalities employed in cancer treatment, most patients receive comprehensive treatment regimens extending beyond pharmacotherapy alone. These non-medication interventions can lead to complications or AEs of differing severity, including:

- Radiotherapy-related: Oral/pharyngeal mucositis, localized pain, swelling/edema, and organ-specific toxicities.
- Interventional procedure-related: Localized pain, fever, nausea/vomiting, and post-embolization syndrome.
- Surgery-related: Pain, hemorrhage, infection, and scar formation.
- Novel therapies (e.g., immunocellular/gene therapy): associated AEs specific to the modality.

Consequently, the implementation of stratified pharmaceutical care for cancer patients requires consideration not only of pathophysiological factors and pharmacotherapy, but also of non-pharmacological interventions such as radiotherapy, interventional procedures, surgery, and other therapies. A comprehensive risk, multidisciplinary risk assessment encompassing all treatment modalities is essential for determining the appropriate level of pharmaceutical care.

The recommended criteria and summarized framework for stratified pharmaceutical care for cancer patients in this consensus are presented in Table 2.

**TABLE 2:** Stratification criteria for pharmaceutical care in adult cancer patients.

Category	Level 1 pharmaceutical care	Level 2 pharmaceutical care	Level 3 pharmaceutical care
Pathophysiological status			
Special physiological conditions			
Fertility plan	Patients with planned gonadotoxic anticancer therapy	​	​
Age	​	​	Age ≥75 years
Weight	​	​	Abnormal body mass index (BMI <18.5 kg/m^2^ or ≥28 kg/m^2^)
Performance status	​	​	ECOG performance status ≥2 or KPS ≤70
Special pathological conditions			
Chronic comorbidities	​	Cancer patients with unstable chronic diseases (e.g., hypertension, diabetes, COPD)	Cancer patients with relatively stable chronic diseases
Infection	Severe infection	Moderate infection	Mild infection
Nutritional status	​	Severe malnutrition (PG-SGA C)	Any of the following: (a) Nutritional risk by NRS2002 score (≥3) (b) Moderate/suspected malnutrition by PG-SGA B (c) Receiving parenteral nutrition or enteral feeding
Pain	​	Opioid-naive patients, those with opioid-related safety risk factors (e.g., renal/hepatic dysfunction, respiratory impairment, sleep apnea, poor performance status), or those with inadequate pain control despite opioid use	​
Liver and renal impairment	Severe impairment	Moderate impairment	Mild impairment
Medication-related factors			
Antineoplastic agents toxicity risk management			
Cardiovascular system	​	Any of the following criteria are met: (a) Use of anthracyclines (e.g., doxorubicin, epirubicin) with high-risk cardiac toxicity, in the presence of cardiac toxicity risk factors (b) Use of antineoplastic agents with moderate-risk cardiac toxicity (e.g., trastuzumab, fluorouracil), in patients with two or more cardiac toxicity risk factors (c) Patients presenting with symptoms such as myasthenia and myositis after the use of immune checkpoint inhibitors, who are suspected of developing having immune-mediated myocarditis	Apply if Level 2 criteria are not met and any of the following criteria are fulfilled: (a) High cumulative doses of anthracyclines (doxorubicin ≥250 mg/m^2^, epirubicin ≥600 mg/m^2^) (b) Sequential administration of anthracyclines followed by trastuzumab (c) Use of antineoplastic agents with potential cardiotoxicity (excluding than anthracyclines) in patients with cardiotoxicity risk factors (d) Use of ICIs (characterized by low incidence but high mortality of cardiotoxicity) in patients with additional risk factors (e.g., combination immunotherapy with PD-1 inhibitor plus CTLA-4 inhibitor, thymic tumors) (e) Use of antineoplastic agents with minimal cardiotoxicity but a high incidence of other cardiovascular toxicities (e.g., hypertension caused by anti-angiogenic agents such as bevacizumab), in patients with risk factors that may exacerbate cardiovascular toxicity
Respiratory system	Any of the following criteria are met: (a) Cumulative exposure dose of bleomycin ≥400 IU (b) Cumulative exposure dose of carmustine ≥1,500 mg/m^2^ (c) Cumulative exposure dose of mitomycin ≥50 mg/m^2^	Any of the following criteria are met in patients with high-risk factors for respiratory toxicity: (a) Cumulative exposure dose of bleomycin <400 IU (b) Cumulative exposure dose of carmustine <1,500 mg/m^2^ (c) Cumulative exposure dose of mitomycin <50 mg/m^2^ (d) Radiotherapy (with a focus on thoracic irradiation) combined with chemotherapy (primarily comprising anthracyclines, paclitaxel, gemcitabine, and pemetrexed)	Any of the following criteria are met: (a) Cumulative exposure dose of bleomycin <400 IU (b) Cumulative exposure dose of carmustine <1,500 mg/m^2^ (c) Cumulative exposure dose of mitomycin <50 mg/m^2^ (d) Radiotherapy (with a focus on thoracic irradiation) combined with chemotherapy (primarily comprising anthracyclines, paclitaxel, gemcitabine, and pemetrexed) (e) Treatment with PD-1/PD-L1 inhibitors, CTLA-4 inhibitors, or BCG (f) Treatment with EGFR-TKIs, ALK inhibitors, ROS1 inhibitors, NTRK inhibitors, RET inhibitors, MET inhibitors, BCR-ABL inhibitors, mTOR inhibitors, or ADCs (g) Targeted therapy with trametinib, rituximab, bevacizumab, pazopanib, duvelisib, avapritinib, gilteritinib, or amivantamab (h) Chemotherapy with cyclophosphamide, lomustine, gemcitabine, or melphalan
Digestive system	​	​	Any of the following criteria are met: (a) Use of antineoplastic agents with a high risk of gastrointestinal toxicity (i.e., a high incidence of gastrointestinal toxicity), along with other high-risk factors (e.g., brain metastases, intestinal obstruction, anxiety disorders, a history of severe vomiting following prior chemotherapy, and irritable bowel syndrome) (b) Use of antineoplastic agents with high hepatotoxic risk, in patients with underlying liver disease and/or concurrent use of other agents with significant hepatotoxic potential (e.g., anti-tuberculosis agents, statins)
Endocrine system	​	Use of antineoplastic agents with a high incidence of endocrine toxicities, combined with one or more high-risk factors for such toxicities (e.g., poorly controlled pre-existing endocrine disorders; radiotherapy to endocrine glands, or a history of endocrine-related adverse events)	Apply if Level 2 criteria are not met and any of the following criteria are fulfilled: (a) Use of ICIs (b) Use of pan-targeted tyrosine kinase inhibitors containing VEGFR (c) Use of ALK inhibitors (d) Use of PI3K, AKT, or mTOR inhibitors (e) Use of bone-modifying agents (e.g., bisphosphonates or denosumab) for ≥2 years (f) Use of endocrine therapy for cancer for ≥5 years (g) Use of VEGFR2 monoclonal antibody (ramucirumab)
Hematological system	​	Any of the following criteria are met: (a) A history of neutropenia during previous cancer treatment, with the current use of high-risk drugs or regimens for neutropenia induction (e.g., MVAC regimen [methotrexate + vinblastine + doxorubicin + cisplatin], FOLFOXIRI regimen [fluorouracil + leucovorin + oxaliplatin + irinotecan], TAC regimen [docetaxel + doxorubicin + cyclophosphamide], and other similar regimens). (b) A history of Grade 3/4 chemotherapy-induced thrombocytopenia (CTIT) during previous cancer treatment, and the current use of drugs or regimens with an incidence of Grade 3/4 CTIT ≥30% (e.g., niraparib, bortezomib, topotecan + cisplatin, and other analogous agents/regimens) (c) A history of severe to life-threatening anemia during prior cancer treatment, with current use of agents such as PARP inhibitors, nivolumab, and others	Apply if Level 2 criteria are not met and any of the following criteria are fulfilled: (a) A history of neutropenia during prior cancer treatment, with current use of intermedium-risk drugs or regimens for neutropenia induction (e.g., AC-T regimen [doxorubicin + cyclophosphamide followed by paclitaxel], docetaxel, irinotecan, and other similar agents/regimens) (b) A history of Grade 3/4 CTIT during prior cancer treatment, with current use of drugs or regimens with an incidence of Grade 3/4 CTIT of 11%–30% (e.g., trastuzumab emtansine, gemcitabine, temozolomide, and other analogous agents/regimens) (c) Use of high-risk drugs or regimens for neutropenia (d) Use of drugs or regimens with an incidence of Grade 3/4 CTIT ≥30% (e) Concurrent use of medium-risk drugs or regimens for neutropenia and drugs or regimens with an incidence of Grade 3/4 CTIT of 11%–30% (f) A history of mild or moderate anemia during previous cancer treatment, with current receipt of agents such as ADCs, platinum-containing agents, PARP inhibitors, immune checkpoint inhibitors, and others
Immune system	Any of the following criteria are met: (a) Patients undergoing hematopoietic stem cell transplantation. (b) Patients undergoing CAR-T cell therapy	Patients with active HBV infection (HBsAg-positive) or inactive HBV infection (HBcAg-positive and HBsAg-negative) receiving anti-B cell agents (e.g., rituximab) are at increased risk of HBV reactivation	Apply if Level 2 criteria are not met and any of the following criteria are fulfilled: (a) Patients with hematological malignancies using anti-B cell drugs, who are at increased the risk of infections (e.g., Pneumocystis pneumonia, cryptococcal infection, Epstein-Barr virus [EBV] infection, and cytomegalovirus [CMV] infection, etc.) (b) Patients with pre-existing autoimmune diseases receiving ICIs
Urinary system	​	Any of the following criteria are met: (a) Use of high-risk or high-dose nephrotoxic antineoplastic agents (e.g., ifosfamide, cisplatin, high-dose cyclophosphamide [single dose ≥1 g/m^2^ or cumulative dose ≥10 g/m^2^], high-dose methotrexate [1–15 g/m^2^]) (b) Use of potentially nephrotoxic systemic antineoplastic in combination with any of the following risk factors: Intravascular volume depletion (e.g., fluid loss, edema); Concurrent nephrotoxic medications (e.g., aminoglycosides, nonsteroidal anti-inflammatory drugs [NSAIDs], proton pump inhibitors [PPIs]); Urinary tract obstruction; Pre-existing or acquired renal disease (c) Use of high-risk nephrotoxic antineoplastic agents combined with radiotherapy targeting the urinary system	Apply if Level 2 criteria are not met and any of the following criteria are fulfilled: (a) A history of urinary system impairment (e.g., chronic kidney disease, bladder dysfunction) receiving potentially nephrotoxic antineoplastic agents (b) Prior exposure to potentially nephrotoxic antineoplastic agents, with current switching to another agent within the same nephrotoxic class (c) Use of potentially nephrotoxic antineoplastic agents (e.g., carboplatin, non-high-dose methotrexate, non-high-dose cyclophosphamide, oxaliplatin, gemcitabine, capecitabine, pemetrexed, irinotecan, bevacizumab, abemaciclib, lenvatinib) combined with radiotherapy targeting the urinary system (d) Development of subclinical renal impairment during treatment (e.g., microalbuminuria, elevated serum creatinine) with ongoing therapy required
Nervous system	​	Any of the following criteria are met: Use of high-risk neurotoxic antineoplastic agents administered at cumulative dose or treatment durations associated with neurotoxicity risk: (a) Cisplatin >350 mg/m^2^ cumulative dose (b) Oxaliplatin 780–850 mg/m^2^ cumulative dose (c) Paclitaxel 250–300 mg/m^2^ cumulative dose (d) Docetaxel >100 mg/m^2^ cumulative dose (e) Vincristine 2–6 mg/m^2^ cumulative dose (f) Bortezomib 16–26 mg/m^2^ cumulative dose Or use of high-risk neurotoxic drugs in combination with any of the following high-risk neurological factors: (a) Recurrent Grade 1–2 neurotoxicity (b) History of Grade ≥3 neurotoxicity (c) Pre-existing neurological disorders (d) Impaired liver/kidney function	Apply if Level 2 criteria are not met and any of the following criteria are fulfilled: (a) Chemotherapy drugs with a >1% incidence of neurotoxicity (b) ADCs containing MMAE as the cytotoxic payload (c) Third-generation ALK tyrosine kinase inhibitors (TKIs) (d) Poly (ADP-ribose) polymerase (PARP) inhibitors (e) Immune checkpoint inhibitors (Widemann and Adamson, 2006) (f) Adoptive cell therapy
Skin and mucosal system	​	Any of the following criteria are met: (a) Patients receiving antineoplastic agents with a high propensity for skin toxicities and complex management (e.g., amivantamab) (b) Patients with a history of Grade 3/4 mucocutaneous toxicity following prior antineoplastic therapy who require re-challenge with the same or analogous agents (c) Combination of agents with overlapping skin toxicity profiles (e.g., capecitabine plus anlotinib, which increases the risk of hand-foot skin toxicity)	Any of the following criteria are met: (a) Patients receiving antineoplastic agents with a higher risk of skin toxicity (e.g., capecitabine, regorafenib, everolimus) in the presence of exacerbating risk factors; or concurrent radiotherapy with paclitaxel, docetaxel, anthracyclines, fluorouracil, or EGFR inhibitors (b) Patients with pre-existing autoimmune skin conditions who are at increased risk of developing or experiencing a relapse of skin reactions when treated with ICIs
Other (insufficient experience in the use of new antineoplastic drugs)	​	Any of the following criteria are met: (a) New antineoplastic agents with severe ADRs (Grade 3–4) with an incidence >30%, as specified in the prescribing information or randomized controlled trials (b) New anti-tumor drugs with warnings in the prescribing information related to ADRs (c) Agents with severe but rare ADRs reported in the drug instructions or literature (d) Agents for the first use in the medical institution (e) Agents with frequent off-label use (f) Agents with a broad target spectrum	​
Antineoplastic agents toxicity management	Life-threatening or disabling ADRs requiring permanent discontinuation	Serious ADRs with medical significance but not immediately life-threatening; may adjust or pause therapy	All other ADRs not listed in Level 1 or 2
Therapeutic drug monitoring (TDM) and pharmacogenomics			
TDM	Patients with drug concentrations outside the target range and with toxicity	Patients with concentrations within the range but with toxicity, or outside the range without toxicity	Patients with concentrations within the range and without toxicity
Pharmacogenomics	​	Patients with abnormal drug metabolism genotypes (ultra-rapid or poor metabolizers)	​
Clinically significant drug-drug interactions (DDIs)	​	Clinically significant drug interactions that cannot be avoided	Clinically significant drug interactions manageable via regimen adjustment
Polypharmacy	​	Patients taking ≥10 non-antineoplastic agents	Patients taking 5–9 non-antineoplastic agents
Special routes of administration or delivery devices	​	Any of the following criteria are met: (a) Cancer patients receiving enteral tube feeding with enteric-coated preparations, sustained-/controlled-release formulations, pharmaceutically incompatible medications, hyperosmolar fluids (>500 mOsm/L), or drugs prone to precipitation (b) Cancer patients with gastrointestinal dysfunction requiring enteral feeding	​
Medication adherence	​	​	Patients with suboptimal medication adherence (e.g., missed doses, incorrect administration, self-adjusted dosing, treatment refusal)
Complex medication issues	​	Cancer patients with complex medication issues requiring collaborative decision-making by ≥ 2 clinical pharmacists from different specialties (e.g., oncology, anti-infectives, enteral/parenteral nutrition)	​
Non-medication therapeutic interventions			
Conventional non-medication interventions	Cancer patients experiencing life-threatening or disabling complications or AEs from non-medication interventions (e.g., radiotherapy, interventional therapy, or surgery)	Cancer patients experiencing severe but non-life-threatening complications or AEs from non-pharmacological interventions (e.g., radiotherapy, interventional therapy, or surgery)	Cancer patients experiencing mild-to-moderate complications or AEs from non-pharmacological interventions (e.g., radiotherapy, interventional therapy, or surgery)
Novel non-medication interventions	Any of the following criteria are met: (a) Patients receiving immunocellular therapies (e.g., CAR-T, TIL) who develop complications (b) Patients undergoing gene therapy, targeted radionuclide therapy (e.g.,177Lu-PSMA), or physical ablation/ photodynamic therapy who develop severe complications or AEs	Any of the following criteria are met: (a) Patients receiving immunocellular therapies (e.g., CAR-T, TIL) who have no complications (b) Patients undergoing gene therapy, targeted radionuclide therapy (e.g.,177Lu-PSMA), or physical ablation/ photodynamic therapy who develop mild-to-moderate complications or AEs	​

## 6 Disscusion

The stratified pharmaceutical care framework proposed in this consensus is designed to address the core challenge of balancing limited pharmacist resources with the complex needs of oncology patients. To ensure this framework translates into tangible clinical value and to systematically measure its implementation quality, quantitative evaluation tools are essential. Furthermore, we propose a set of key performance indicators (KPIs) derived from published evidence (de Souza et al., 2024; Magedanz et al., 2024; Muluneh et al., 2024), which are tailored to objectively assess the impact of pharmacist-led interventions on medication safety, treatment adherence, and patient outcomes—particularly targeting the high-risk oncology patients identified through this consensus. These KPIs are as follows:

- Early Detection Rate of Severe Adverse Drug Reactions (ADRs)

Percentage of Grade ≥3 ADRs (CTCAE v5.0) detected by proactive pharmacist monitoring before they precipitate an emergency-department visit, hospitalisation, or unplanned treatment modification. An increasing rate signals enhanced vigilance and timeliness of intervention.

- Adherence Improvement in High-Risk Cohorts
  Change in adherence, measured with Medication Possession Ratio (MPR) or Proportion of Days Covered (PDC), between baseline and 3 months after intensive pharmacist intervention in patients at high risk of non-adherence (e.g., complex oral regimens, cognitive impairment, socioeconomic barriers).
- Appropriateness of Dose Adjustments in Organ Dysfunction
  Proportion of patients with documented renal or hepatic impairment whose anticancer regimen is dose-adjusted in accordance with current labeling or consensus guidelines.
- Intervention Rate for Clinically Significant DDIs
  Percentage of flagged, clinically significant DDIs for which a documented pharmacist recommendation (dose change, alternative therapy, additional monitoring, or prescriber consultation) is accepted and implemented.
- Patient Satisfaction with Oncology Pharmaceutical Care

Percentage of respondents scoring ≥4 on a 5-point Likert scale in a validated survey (e.g., Patient Satisfaction with Pharmaceutical Care Questionnaire) evaluating communication, accessibility, counselling quality, and perceived support during treatment.

These KPIs provide a structured, data-driven approach to evaluating the effectiveness of the stratified care model, facilitating continuous quality improvement and demonstrating the value of clinical pharmacy services within oncology care teams.

To enhance implementation and scalability, healthcare institutions should integrate the stratification criteria within Hospital Information Systems (HIS), Electronic Medical Records (EMR), or Clinical Decision Support Systems (CDSS). Automating the identification and categorization process reduces manual workload, improves accuracy, and allows pharmacists to focus on high-value clinical activities. Moreover, digital integration facilitates real-time data collection for KPI monitoring and real-world evidence (RWE) generation, which will be essential for future optimization.

Looking forward, several strategic priorities should guide the evolution of this framework. First, multi-center studies are needed to generate robust RWE and verify the system’s impact on clinically meaningful endpoints, such as reduced ADR-related hospitalizations and improved survival. Second, regular updates to the stratification criteria will be required to keep pace with rapidly evolving cancer therapeutics. Third, integrating artificial intelligence (AI) and predictive analytics could transition the framework from static rules to dynamic, individualized risk prediction (Naderian et al., 2025; Huang et al., 2025; Abdelmohsen and Al-Jabri, 2025). Finally, tailoring and standardizing the framework across healthcare settings—particularly in resource-limited regions—will be essential for promoting equitable, high-quality oncology pharmaceutical care.

In summary, while this consensus provides a foundational framework, its full potential will be realized through digital implementation, rigorous outcome assessment, and continuous adaptation to the evolving oncology landscape.

## 7 Conclusion

This consensus proposes an evidence- and experience-based stratification framework for cancer patients pharmaceutical care. The adoption of this structured classification system is expected to optimize resource allocation, enhance the quality and consistency of clinical pharmacy services, and ultimately improve patient safety and therapeutic outcomes.

This work represents an important step toward advancing patient-centered pharmaceutical care within China’s oncology setting. The methodology and principles underlying this framework are broadly adaptable and may serve as a reference for institutions or healthcare systems seeking to establish their own stratified approaches to oncology pharmacy practice. Future efforts should emphasize broader adoption, real-world performance assessment, and cross-institutional collaboration to further enhance its applicability and long-term impact.

## Data Availability

The original contributions presented in the study are included in the article/Supplementary Material, further inquiries can be directed to the corresponding authors.
