# Supplementary material for: Stratification system for pharmaceutical care in cancer patients: Chinese expert consensus
Source: Front Pharmacol. 2026 Feb 16;16:1707229. doi: 10.3389/fphar.2025.1707229 (PMC12950709; doi:10.3389/fphar.2025.1707229)
Supplement: Supplementary file 1 [file Supplementaryfile1.docx]

**Supplementary Material 1**

**Detailed Methodology for Consensus Development**

**1 Evidence Review**

**1.1Materials and Methods**

***1.1.1 Inclusion and Exclusion Criteria***

Literature was retrieved from PubMed, Embase, and The Cochrane Library (for English publications) as well as CNKI, VIP, CBM, and Wanfang Data (for Chinese publications). Studies meeting the following criteria were included: (1) Chinese and English publications explicitly mentioning pharmaceutical monitoring indicators or influencing factors; (2) study types covering official documents, guidelines, systematic reviews, narrative reviews, and cross-sectional studies.

***1.1.2 Retrieval Strategy***

A systematic search was performed using "pharmaceutical care" and "indicator" as keywords to identify English-language literature published from database inception to December 31, 2024. A separate systematic search was conducted for Chinese-language literature during the same period, with "pharmaceutical care" and "stratification" as the keywords. For instance, the retrieval strategy for PubMed is presented in Table S1.

**Table S1 The search formula of PubMed**

| **Search** **number** | **Query** |
| --- | --- |
| #1 | Search: pharmaceutical care [MeSH Terms] |
| #2 | Search: ((pharmaceutical care [Title/Abstract]) OR (Pharmaceutical Monitoring [Title/Abstract])) |
| #3 | #1 OR #2 |
| #4 | Search: indicator [Title/Abstract] |
| #5 | #3 AND #4 |

***1.1.3 Literature Screening and Data Extraction***

Two researchers initially screened titles and abstracts to exclude non-eligible studies, then independently conducted full-text reviews of potentially eligible studies. Uncertain cases were discussed to determine inclusion. Finally, two researchers independently extracted information from the included studies in a blinded manner. Key data extracted included: (1) Basic study details (title, authors, publication year, journal); (2) Study design; (3) Content relevant to the research theme. The JBI Critical Appraisal Checklist for Qualitative Research was utilized to assess the quality of studies meeting the inclusion and exclusion criteria. Studies with quality scores ≥6 were selected, and data extraction was performed using the JBI System for the Unified Management, Assessment, and Review of Information.

***1.4 Data Synthesis***

Using a thematic synthesis approach to summarize key information, we ultimately identified pharmaceutical monitoring indicators and influencing factors.

**1.2 Results**

***1.2.1 Basic Information of Included Studies***

This study included a total of 6,328 documents (1,893 Chinese documents and 4,435 English documents). After screening and excluding 6,234 studies lacking sufficient evidence, 94 studies were ultimately included (shown in Figure S1). See Table S2 and S3 for details.

**Figure S1 Literature Screening Process for Systematic Reviews**

***1.2.2 Current Status of Pharmaceutical Monitoring Indicators***

The systematic review identified 94 relevant studies, including 36 addressing pharmaceutical care indicators and 58 describing factors influencing pharmaceutical care (shown in Table S2). Existing pharmaceutical care indicators primarily exhibit two issues: (1) incomplete coverage; and (2) unsuitability for cancer patients. Therefore, there is an urgent need to establish a pharmaceutical care indicator system tailored to cancer patients.

***1.2.3 Data Analysis and Synthesis***

Analysis of pharmaceutical monitoring indicators and influencing factors categorizes indicators into three dimensions: physiological and pathological states, pharmacological interventions, and non-pharmacological interventions. See Table S4 for details.

**Table S2 Inclusion in the Research Overview**

| **Author/Year** | **Population** | **Elements of Guardianship Classification** |
| --- | --- | --- |
| Alshaikhmubarak FQ, 2025 ^[1]^ | Chronic disease patients | Liver and kidney function, Maternity Protection, Medication adherence, Multiple medications, Age |
| Chen Xuefeng, 2025 ^[2]^ | Diabetes patients | Health literacy |
| Doublet S, 2025 ^[3]^ | Cancer patients | Physical Strength Score |
| Huang Jiaqiao, 2025 ^[4]^ | Cancer patients | Medication adherence, ADR Management, Medication Education |
| Luo W, 2025 ^[5]^ | Cancer patients | Cancer Pain Management |
| Mohammed AH, 2025 ^[6]^ | Cancer patients | Infection, antibiotic use |
| Muluneh B, 2025 ^[7]^ | Cancer patients | Medication adherence |
| Sun Yan, 2025 ^[8]^ | Children with epilepsy | Complexity of the condition, stability of the condition, Liver and kidney function |
| Yang Y, 2025 ^[9]^ | Ovarian Cancer patients | Nutritional Support |
| Bansal N, 2024 ^[10]^ | Cancer patients | Cancer Pain Management, Drug-drug interactions, Disease state |
| Canning ML, 2024 ^[11]^ | Chronic disease patients | Pharmacotherapy Management |
| Cao Yong, 2024 ^[12]^ | Diabetes patients | Organ function, Comorbidities, Multiple medications, Treatment Plan, Severity of Disease, ADR Management |
| de Souza JFF, 2024 ^[13]^ | Chronic disease patients | Pharmacotherapy Management |
| Gossery C, 2024 ^[14]^ | Cancer patients | Drug-drug interactions, Pharmacovigilance |
| Lian Yufei, 2024 ^[15]^ | Hospitalized patients with chronic airway disease | Comorbidities, Liver and kidney function, Multiple medications, Medication adherence, ADR Management |
| Maezawa T, 2024 ^[16]^ | Cancer patients | Maternity Protection |
| Schönenberger N, 2024 ^[17]^ | Chronic disease patients | Multiple medications, Medication adherence, Comorbidities, Drug-drug interactions |
| Shrestha S, 2024 ^[18]^ | Cancer patients | Cancer Pain Management |
| Xue Zhi, 2024 ^[19]^ | Patients with atrial fibrillation | Age, Disease state, Liver and kidney function, Comorbidities, Multiple medications, Medication adherence |
| Yu Yufa, 2024 ^[20]^ | Individuals with depressive disorders | Disease state, Multiple medications, Liver and kidney function, ADR Management |
| Zhang Ruobin, 2024 ^[21]^ | Patients with COPD | Severity of Disease, Medication adherence, Comorbidities, Multiple medications |
| Zhao Yanping, 2024 ^[22]^ | Patients with lung cancer undergoing perioperative care | Preoperative VAS score, surgical procedure, smoking history, anxiety level, Comorbidities, Age, BMI |
| Zhou Huan, 2024 ^[23]^ | Patients with COPD | Pulmonary function, types of medication |
| Betts AC, 2023 ^[24]^ | Cancer patients | Comorbidities, Multiple medications, Cancer Pain Management |
| Chang CE, 2023 ^[25]^ | Chronic disease patients | Pathophysiological basis, organ dysfunction, ADR Management |
| Chen Jiahui, 2023 ^[26]^ | Diabetes patients | Liver and kidney function, Age, Multiple medications, Medication adherence, Severity of Disease |
| Gong Y, 2023 ^[27]^ | Chronic disease patients | Medication adherence, ADR Management |
| Gong Ying, 2023 ^[28]^ | Patients with stroke-associated pneumonia | Disease state, Severity of Disease, Comorbidities, Multiple medications, Liver and kidney function |
| Jiang Huanhuan, 2023 ^[29]^ | Urology Perioperative Patients | Age, Liver and kidney function, ADR Management, Multiple medications, Pregnancy and lactation, history of allergies, drug interactions, special administration routes, potential medication risks, Disease state |
| Jost N, 2023 ^[30]^ | Cancer patients | Nutritional Support |
| Qin Qiong, 2023 ^[31]^ | Diabetes patients | Organ function, Comorbidities, Multiple medications, Medication adherence, Treatment Plan, Severity of Disease, ADR Management |
| Wang Mengmeng, 2023 ^[32]^ | Children with generalized myasthenia gravis | Severity of Disease |
| Wei Wei, 2023 ^[33]^ | Heart failure patients | Organ function, Multiple medications, Medication adherence, Treatment Plan, Severity of Disease |
| Xie H, 2023 ^[34]^ | Cancer patients | Cancer Pain Management |
| Zhang Chunge, 2023 ^[35]^ | Patients with systemic lupus erythematosus | Clinical Disease Severity Classification, Knowledge Dimension, Belief Dimension, Behavior Dimension |
| Bates N, 2022 ^[36]^ | Cancer patients | Drug-drug interactions, Cancer Pain Management |
| Botelho SF, 2022 ^[37]^ | Chronic disease patients | Drug-drug interactions |
| Chai Dongyan, 2022 ^[38]^ | Drug-dependent patients | The difficulty level of drug-related issues |
| Chen Yan, 2022 ^[39]^ | Patients on dual antiplatelet therapy | Age, platelet count, peptic ulcer/H. pylori infection, combination therapy, ADR Management, history of thrombosis/bleeding |
| Dai Mengfei, 2022 ^[40]^ | Patients receiving warfarin anticoagulation therapy | Severity of Disease, Age, Liver and kidney function, Comorbidities, Multiple medications, ADR Management, Relevant medical history |
| En-Nasery-de Heer S, 2022 ^[41]^ | Breast cancer patients | Medication adherence, ADR Management |
| Fang Yuan, 2022 ^[42]^ | Chronic disease patients | Disease state, infection, cancer pain management, coagulation function, hepatic and renal function, parenteral nutrition, polypharmacy, ADR management |
| He Peihua, 2022 ^[43]^ | Schizophrenia patients | Disease state, Comorbidities, Liver and kidney function, Number of oral medications, Medication adherence, ADR Management |
| Hoegy D, 2022 ^[44]^ | Chronic disease patients | Comorbidities, ADR Management, Weight, age |
| Jiang Jie, 2022 ^[45]^ | Chronic disease patients | Severity of Disease, Liver and Kidney Function, Respiratory Function, Special Pathophysiology, Special Pharmacotherapy, ADR Management, Therapeutic Drug Monitoring |
| Lockman K, 2022 ^[46]^ | Cancer patients | Medication Management, Clinical Care, Cancer Pain Management, ADR Management |
| Mashni OK, 2022 ^[47]^ | Cancer patients | Pharmacotherapy Management |
| National Cancer Center, 2022 ^[48]^ | Chronic disease patients | ADR Management |
| Pirolli AV, 2022 ^[49]^ | breast cancer patients | Medication adherence, Individualized medication administration |
| Sato N, 2022 ^[50]^ | Chronic disease patients | Special Administration Routes, Medication adherence, Drug-drug interactions, Patient-related factors |
| Shawahna R, 2022 ^[51]^ | Chronic disease patients | Personalized drug administration |
| Wang Liman, 2022 ^[52]^ | Patients receiving warfarin anticoagulation therapy | Severity of Disease, Age, Liver and kidney function, Comorbidities, Multiple medications, Medication adherence, ADR Management, Relevant medical history |
| Li Shuyue, 2021 ^[53]^ | Chronic disease patients | Liver and kidney function, ADR Management, Medication adherence, Special populations |
| Moukafih B, 2021 ^[54]^ | Chronic disease patients | Drug restructuring, Pharmacotherapy Management |
| Bakker T, 2020 ^[55]^ | Chronic disease patients | Drug-drug interactions |
| Chen Jie, 2020 ^[56]^ | Chronic disease patients | Medication adherence, Disease state |
| Chen Min, 2020 ^[57]^ | Chronic disease patients | Nutritional Support |
| Pamulapati LG, 2020 ^[58]^ | Diabetes patients | Medication history, Components of physical examination, Patient education |
| Przybylski DJ, 2020 ^[59]^ | Chronic disease patients | Pharmacogenomics-Based Personalized Drug Delivery |
| Shawahna R, 2020 ^[60]^ | Chronic disease patients | Pharmacotherapy Management |
| Vu K, 2020 ^[61]^ | Cancer patients | ADR Management |
| Boşnak AS, 2019 ^[62]^ | Cancer patients | Drug restructuring, ADR Management |
| Boutin K, 2019 ^[63]^ | Patients with kidney disease | Renal function, Medication adherence, ADR Management |
| Hou Jiqiu, 2019 ^[64]^ | Patients with COPD | Pulmonary function, clinical symptoms |
| Hu Wenjuan, 2019 ^[65]^ | Children with asthma | Severity of Disease |
| Mairead McGrattan, 2019 ^[66]^ | Dementia patients | Medication adherence, Drug-drug interactions, ADR Management |
| Mubarak N, 2019 ^[67]^ | Chronic disease patients | Drug-drug interactions，Medication adherence |
| Patel H, 2019 ^[68]^ | Prostate cancer patients | Medication adherence, Improved quality of life, satisfaction |
| Qin Qiong, 2019 ^[69]^ | Chronic disease patients | Disease state, Liver and kidney function, cardiac function, infection, Comorbidities, combination therapy, Multiple medications, Medication adherence, Treatment Plan, |
| Zeh S, 2019 ^[70]^ | Chronic disease patients | Personalized drug administration |
| Zhang Yongli, 2019 ^[71]^ | Postoperative neurosurgical patients | Treatment Plan, Multiple medications, Liver and kidney function, Special Administration Routes |
| Zuo Yun, 2019 ^[72]^ | Chronic disease patients | Disease state, Special populations, Number of Drug Categories |
| Cheng Kang, 2018 ^[73]^ | Patients with COPD | Pulmonary function, Multiple medications |
| Tian Weiwei, 2018 ^[74]^ | Chronic disease patients | Liver and kidney function, Disease state, Relevant medical history, ADR Management, Medication adherence, Special populations, Multidisciplinary Rounds |
| Watanabe T, 2018 ^[75]^ | Cervical cancer patients | Medication adherence |
| Colombo LRP, 2017 ^[76]^ | Cancer patients | Drug restructuring, ADR Management |
| Gavila J, 2017 ^[77]^ | breast cancer patients | Cardiac Toxicity Management |
| Moulin SM, 2017 ^[78]^ | Patients with chronic leukemia | Medication adherence |
| Shen Xiaoyun, 2016 ^[79]^ | ICU patients | Liver and kidney function, Blood pressure, Multiple medications, Cardiac Function and Arrhythmia |
| Terada T, 2016 ^[80]^ | Cancer patients | Pharmacokinetics-Based Personalized Dosing |
| Bu Yishan, 2015 ^[81]^ | Chronic disease patients | Liver and kidney function, Disease state, Multiple medications, ADR Management, TDM |
| Gao Ning, 2015 ^[82]^ | Cancer patients | age, Liver and kidney function, Multiple medications, Drug-drug interactions, Medication Risks, ADR Management, Treatment Plan, Comorbidities, Disease state |
| Mahabaleshwarkar R, 2015 ^[83]^ | Breast cancer patients | Comorbidities |
| Dreesen M, 2013 ^[84]^ | Cancer patients | Nutritional Support |
| Fiß T, 2013 ^[85]^ | Dementia patients | Medication adherence, Drug-drug interactions, ADR Management |
| Ross LA, 2012 ^[86]^ | Chronic disease patients | Pharmacotherapy Management |
| Wierenga PC, 2011 ^[87]^ | Old patients | Pharmacotherapy Management |
| Lai PS, 2010 ^[88]^ | postmenopausal women | Medication adherence |
| Chan A, 2009 ^[89]^ | Cancer patients | Drug-drug interactions, Number of Drug Categories |
| Scarpace SL, 2009 ^[90]^ | Patients with head and neck tumors | ADR Management |
| Setoguchi S, 2008 ^[91]^ | Cancer patients | Cancer Pain Management |
| Hayakawa T, 2005 ^[92]^ | Lung cancer patients | ADR Management |
| Hermansen-Kobulnicky CJ, 2004 ^[93]^ | Cancer patients | ADR Management |
| Edington J, 1999 ^[94]^ | Cancer patients | Nutritional Support |

**Table S3 Quality assessment results of the studies included**

| **Author/Year** | **Item 1** | **Item 2** | **Item 3** | **Item 4** | **Item 5** | **Item 6** | **Item 7** | **Item 8** | **Item 9** | **Item 10** | **Quality Score** |
| --- | --- | --- | --- | --- | --- | --- | --- | --- | --- | --- | --- |
| Alshaikhmubarak FQ, 2025 ^[1]^ | Y | Y | Y | Y | Y | Y | Y | Y | Y | Y | 10 |
| Chen Xuefeng, 2025 ^[2]^ | Y | Y | Y | Y | Y | Y | Y | Y | Y | Y | 10 |
| Doublet S, 2025 ^[3]^ | Y | Y | Y | Y | Y | Y | Y | Y | Y | Y | 10 |
| Huang Jiaqiao, 2025 ^[4]^ | Y | Y | Y | Y | Y | Y | Y | Y | Y | Y | 10 |
| Luo W, 2025 ^[5]^ | Y | Y | Y | Y | Y | Y | Y | Y | Y | Y | 10 |
| Mohammed AH, 2025 ^[6]^ | Y | Y | Y | Y | Y | Y | Y | Y | Y | Y | 10 |
| Muluneh B, 2025 ^[7]^ | Y | Y | Y | Y | Y | Y | Y | Y | Y | Y | 10 |
| Sun Yan, 2025 ^[8]^ | Y | Y | Y | Y | Y | Y | Y | Y | Y | Y | 10 |
| Yang Y, 2025 ^[9]^ | Y | Y | Y | Y | Y | Y | Y | Y | Y | Y | 10 |
| Bansal N, 2024 ^[10]^ | Y | Y | Y | Y | Y | Y | Y | Y | Y | Y | 10 |
| Canning ML, 2024 ^[11]^ | Y | Y | Y | Y | Y | Y | Y | Y | Y | Y | 10 |
| Cao Yong, 2024 ^[12]^ | Y | Y | Y | Y | Y | Y | Y | Y | Y | Y | 10 |
| de Souza JFF, 2024 ^[13]^ | Y | Y | Y | Y | Y | Y | Y | Y | Y | Y | 10 |
| Gossery C, 2024 ^[14]^ | Y | Y | Y | Y | Y | Y | Y | Y | Y | Y | 10 |
| Lian Yufei, 2024 ^[15]^ | Y | Y | Y | Y | Y | Y | Y | Y | Y | Y | 10 |
| Maezawa T, 2024 ^[16]^ | Y | Y | Y | Y | Y | Y | Y | Y | Y | Y | 10 |
| Schönenberger N, 2024 ^[17]^ | Y | Y | Y | Y | Y | Y | Y | Y | Y | Y | 10 |
| Shrestha S, 2024 ^[18]^ | Y | Y | Y | Y | Y | Y | Y | Y | Y | Y | 10 |
| Xue Zhi, 2024 ^[19]^ | Y | Y | Y | Y | Y | Y | Y | Y | Y | Y | 10 |
| Yu Yufa, 2024 ^[20]^ | Y | Y | Y | Y | Y | Y | Y | Y | Y | Y | 10 |
| Zhang Ruobin, 2024 ^[21]^ | Y | Y | Y | Y | Y | Y | Y | Y | Y | Y | 10 |
| Zhao Yanping, 2024 ^[22]^ | Y | Y | Y | Y | Y | Y | Y | Y | Y | Y | 10 |
| Zhou Huan, 2024 ^[23]^ | Y | Y | Y | Y | Y | Y | Y | Y | Y | Y | 10 |
| Betts AC, 2023 ^[24]^ | Y | Y | Y | Y | Y | Y | Y | Y | Y | Y | 10 |
| Chang CE, 2023 ^[25]^ | Y | Y | Y | Y | Y | Y | Y | Y | Y | Y | 10 |
| Chen Jiahui, 2023 ^[26]^ | Y | Y | Y | Y | Y | Y | Y | Y | Y | Y | 10 |
| Gong Y, 2023 ^[27]^ | Y | Y | Y | Y | Y | Y | Y | Y | Y | Y | 10 |
| Gong Ying, 2023 ^[28]^ | Y | Y | Y | Y | Y | Y | Y | Y | Y | Y | 10 |
| Jiang Huanhuan, 2023 ^[29]^ | Y | Y | Y | Y | Y | Y | Y | Y | Y | Y | 10 |
| Jost N, 2023 ^[30]^ | Y | Y | Y | Y | Y | Y | Y | Y | Y | Y | 10 |
| Qin Qiong, 2023 ^[31]^ | Y | Y | Y | Y | Y | Y | Y | Y | Y | Y | 10 |
| Wang Mengmeng, 2023 ^[32]^ | Y | Y | Y | Y | Y | Y | Y | Y | Y | Y | 10 |
| Wei Wei, 2023 ^[33]^ | Y | Y | Y | Y | Y | Y | Y | Y | Y | Y | 10 |
| Xie H, 2023 ^[34]^ | Y | Y | Y | Y | Y | Y | Y | Y | Y | Y | 10 |
| Zhang Chunge, 2023 ^[35]^ | Y | Y | Y | Y | Y | Y | Y | Y | Y | Y | 10 |
| Bates N, 2022 ^[36]^ | Y | Y | Y | Y | Y | Y | Y | Y | Y | Y | 10 |
| Botelho SF, 2022 ^[37]^ | Y | Y | Y | Y | Y | Y | Y | Y | Y | Y | 10 |
| Chai Dongyan, 2022 ^[38]^ | Y | Y | Y | Y | Y | Y | Y | Y | Y | Y | 10 |
| Chen Yan, 2022 ^[39]^ | Y | Y | Y | Y | Y | Y | Y | Y | Y | Y | 10 |
| Dai Mengfei, 2022 ^[40]^ | Y | Y | Y | Y | Y | Y | Y | Y | Y | Y | 10 |
| En-Nasery-de Heer S, 2022 ^[41]^ | Y | Y | Y | Y | Y | Y | Y | Y | Y | Y | 10 |
| Fang Yuan, 2022 ^[42]^ | Y | Y | Y | Y | Y | Y | Y | Y | Y | Y | 10 |
| He Peihua, 2022 ^[43]^ | Y | Y | Y | Y | Y | Y | Y | Y | Y | Y | 10 |
| Hoegy D, 2022 ^[44]^ | Y | Y | Y | Y | Y | Y | Y | Y | Y | Y | 10 |
| Jiang Jie, 2022 ^[45]^ | Y | Y | Y | Y | Y | Y | Y | Y | Y | Y | 10 |
| Lockman K, 2022 ^[46]^ | Y | Y | Y | Y | Y | Y | Y | Y | Y | Y | 10 |
| Mashni OK, 2022 ^[47]^ | Y | Y | Y | Y | Y | Y | Y | Y | Y | Y | 10 |
| National Cancer Center, 2022 ^[48]^ | Y | Y | Y | Y | Y | Y | Y | Y | Y | Y | 10 |
| Pirolli AV, 2022 ^[49]^ | Y | Y | Y | Y | Y | Y | Y | Y | Y | Y | 10 |
| Sato N, 2022 ^[50]^ | Y | Y | Y | Y | Y | Y | Y | Y | Y | Y | 10 |
| Shawahna R, 2022 ^[51]^ | Y | Y | Y | Y | Y | Y | Y | Y | Y | Y | 10 |
| Wang Liman, 2022 ^[52]^ | Y | Y | Y | Y | Y | Y | Y | Y | Y | Y | 10 |
| Li Shuyue, 2021 ^[53]^ | Y | Y | Y | Y | Y | Y | Y | Y | Y | Y | 10 |
| Moukafih B, 2021 ^[54]^ | Y | Y | Y | Y | Y | Y | Y | Y | Y | Y | 10 |
| Bakker T, 2020 ^[55]^ | Y | Y | Y | Y | Y | Y | Y | Y | Y | Y | 10 |
| Chen Jie, 2020 ^[56]^ | Y | Y | Y | Y | Y | Y | Y | Y | Y | Y | 10 |
| Chen Min, 2020 ^[57]^ | Y | Y | Y | Y | Y | Y | Y | Y | Y | Y | 10 |
| Pamulapati LG, 2020 ^[58]^ | Y | Y | Y | Y | Y | Y | Y | NA | Y | Y | 9 |
| Przybylski DJ, 2020 ^[59]^ | Y | Y | Y | Y | Y | Y | Y | Y | Y | Y | 10 |
| Shawahna R, 2020 ^[60]^ | Y | Y | Y | Y | Y | Y | Y | Y | Y | Y | 10 |
| Vu K, 2020 ^[61]^ | Y | Y | Y | Y | Y | Y | Y | Y | Y | Y | 10 |
| Boşnak AS, 2019 ^[62]^ | Y | Y | Y | Y | Y | Y | Y | Y | Y | Y | 10 |
| Boutin K, 2019 ^[63]^ | Y | Y | Y | Y | Y | Y | Y | Y | Y | Y | 10 |
| Hou Jiqiu, 2019 ^[64]^ | Y | Y | Y | Y | Y | Y | Y | Y | Y | Y | 10 |
| Hu Wenjuan, 2019 ^[65]^ | Y | Y | Y | Y | Y | Y | Y | Y | Y | Y | 10 |
| Mairead McGrattan, 2019 ^[66]^ | Y | Y | Y | Y | Y | Y | Y | Y | Y | Y | 10 |
| Mubarak N, 2019 ^[67]^ | Y | Y | Y | Y | Y | Y | Y | Y | Y | Y | 10 |
| Patel H, 2019 ^[68]^ | Y | Y | Y | Y | Y | Y | Y | Y | Y | Y | 10 |
| Qin Qiong, 2019 ^[69]^ | Y | Y | Y | Y | Y | Y | Y | Y | Y | Y | 10 |
| Zeh S, 2019 ^[70]^ | Y | Y | Y | Y | Y | Y | Y | Y | Y | Y | 10 |
| Zhang Yongli, 2019 ^[71]^ | Y | Y | Y | Y | Y | Y | Y | Y | Y | Y | 10 |
| Zuo Yun, 2019 ^[72]^ | Y | Y | Y | Y | Y | Y | Y | Y | Y | Y | 10 |
| Cheng Kang, 2018 ^[73]^ | Y | Y | Y | Y | Y | Y | Y | Y | Y | Y | 10 |
| Tian Weiwei, 2018 ^[74]^ | Y | Y | Y | Y | Y | Y | Y | Y | Y | Y | 10 |
| Watanabe T, 2018 ^[75]^ | Y | Y | Y | Y | Y | Y | Y | Y | Y | Y | 10 |
| Colombo LRP, 2017 ^[76]^ | Y | Y | Y | Y | Y | Y | Y | Y | Y | Y | 10 |
| Gavila J, 2017 ^[77]^ | Y | Y | Y | Y | Y | Y | Y | Y | Y | Y | 10 |
| Moulin SM, 2017 ^[78]^ | Y | Y | Y | Y | Y | Y | Y | Y | Y | Y | 10 |
| Shen Xiaoyun, 2016 ^[79]^ | Y | Y | Y | Y | Y | Y | Y | Y | Y | Y | 10 |
| Terada T, 2016 ^[80]^ | Y | Y | Y | Y | Y | Y | Y | Y | Y | Y | 10 |
| Bu Yishan, 2015 ^[81]^ | Y | Y | Y | Y | Y | Y | Y | Y | Y | Y | 10 |
| Gao Ning, 2015 ^[82]^ | Y | Y | Y | Y | Y | Y | Y | Y | Y | Y | 10 |
| Mahabaleshwarkar R, 2015 ^[83]^ | Y | Y | Y | Y | Y | Y | NS | NA | Y | Y | 8 |
| Dreesen M, 2013 ^[84]^ | Y | Y | Y | Y | Y | Y | Y | Y | Y | Y | 10 |
| Fiß T, 2013 ^[85]^ | Y | Y | Y | Y | Y | Y | Y | Y | Y | Y | 10 |
| Ross LA, 2012 ^[86]^ | Y | Y | Y | Y | Y | Y | Y | Y | Y | Y | 10 |
| Wierenga PC, 2011 ^[87]^ | Y | Y | Y | Y | Y | Y | Y | Y | Y | Y | 10 |
| Lai PS, 2010 ^[88]^ | Y | Y | Y | Y | Y | Y | Y | Y | Y | Y | 10 |
| Chan A, 2009 ^[89]^ | Y | Y | Y | Y | Y | Y | Y | Y | Y | Y | 10 |
| Scarpace SL, 2009 ^[90]^ | Y | Y | Y | Y | Y | Y | NS | NA | Y | Y | 8 |
| Setoguchi S, 2008 ^[91]^ | Y | Y | Y | Y | Y | Y | Y | Y | Y | Y | 10 |
| Hayakawa T, 2005 ^[92]^ | Y | Y | Y | Y | Y | Y | Y | Y | Y | Y | 10 |
| Hermansen-Kobulnicky CJ, 2004 ^[93]^ | Y | Y | Y | Y | Y | Y | Y | Y | Y | Y | 10 |
| Edington J, 1999 ^[94]^ | Y | Y | Y | Y | Y | Y | NS | NA | NA | Y | 7 |

Note: Item 1. Is there congruity between the stated philosophical perspective and the research methodology? Item 2. Is there congruity between the research methodology and the research question or objectives? Item 3. Is there congruity between the research methodology and the methods used to collect data? Item 4. Is there congruity between the research methodology and the representation and analysis of data? Item 5. Is there congruity between the research methodology and the interpretation of results? Item 6. Is there a statement locating the researcher culturally or theoretically? Item 7. Is the influence of the researcher on the research, and vice- versa, addressed? Item 8. Are participants, and their voices, adequately represented? Item 9. Is the research ethical according to current criteria or, for recent studies, and is there evidence of ethical approval by an appropriate body? Item 10. Do the conclusions drawn in the research report flow from the analysis, or interpretation, of the data? Y: Yes, N: No, NS: Not sure, NA: Not applicable

**Table S4 Indicators for Pharmaceutical Care By Systematic Review**

| **Primary indicator** | **Secondary Indicators** | **Keywords** |
| --- | --- | --- |
| Pathophysiological Status | Special Physiological Conditions | Fertility plan |
|  |  | Age |
|  |  | Weight |
|  |  | Performance status |
|  | Special Pathological Conditions | Chronic comorbidities |
|  |  | Infection |
|  |  | Nutritional status |
|  |  | Pain |
|  |  | Liver and kidney function |
|  |  | Cardiac function |
|  |  | Smoking History |
| Pharmacotherapy Status | Risk Level of Antineoplastic Drug Use | / |
|  | Anti-tumor Drug Toxicity Risk Management | / |
|  | Monitoring of Personalized Drug Therapy | TDM |
|  |  | Pharmacogenomics |
|  | Polypharmacy | Number of drug varieties |
|  | Clinically Significant Drug-Drug Interactions | / |
|  | Special Routes of Administration or Delivery Devices | / |
|  | Medication Adherence | / |
|  | Coagulation Function and Thrombosis/Bleeding History | / |
|  | Disease State |  |
|  | Tumor Stage | / |
| Other Therapeutic Interventions | Conventional Non-Drug Interventions | / |
|  | Novel Non-Drug Interventions | / |

**2 Cross-sectional Survey**

From January 1 to March 31, 2025, a consensus panel distributed electronic questionnaires to oncology pharmacists at hospitals of various levels or specialized oncology hospitals across the country. The survey content included participants’ basic information, their level of agreement with the graded indicators for oncology pharmaceutical care, and suggestions for revisions. A total of 215 questionnaires were distributed, with 174 valid responses collected. Among the respondents, 126 were clinical pharmacists and 58 were clinical physicians. Deputy senior title or higher accounted for 55.17%, and 71.84% were from tertiary hospitals. For details, refer to Table S5.

Overall, 85.06% of participants fully agreed with these indicators. Six participants disagreed, arguing that the stratified indicators for pharmaceutical care in cancer patients were overly complex. Another 11 participants partially agreed and put forward revision suggestions, with the key points as follows: (1) Eight participants proposed classifying the "Anti-tumor Drug Toxicity Risk Management" section by organ systems, while three suggested classifying Toxicity Risk Management by anti-tumor drug types; (2) Four participants recommended removing "Coagulation Function and Thrombosis/Bleeding History"; (3) Two participants suggested adding mental and psychological disorders as an indicator; (4) Five participants indicated that some complex clinical pharmacotherapy issues requiring collaboration among multi-professional clinical pharmacists cannot be covered by these indicators, and suggested adding an "Other" category for supplementation.

**Table S5 Characteristics of the respondents (N=174)**

| **Characters** | **Category** | **n (%)** |
| --- | --- | --- |
| Sex | Female | 121 (69.5) |
|  | Male | 53 (30.5) |
| Age, years | < 30 | 48 (27.6) |
|  | 30 - 49 | 104 (59.8) |
|  | ≥ 50 | 22 (12.6) |
| Professional Title | Junior Level | 31 (17.8) |
|  | Middle Level | 88 (50.6) |
|  | Senior Level and Above | 55 (31.6) |
| Type of Hospital | Tertary | 131 (75.3) |
|  | Secondary | 40 23.0) |
|  | Primary | 3 (1.7) |
| Highest Education Level | Bachelor's or Lower | 59 (33.9) |
|  | Master's | 92 (52.9) |
|  | PhD | 23 (13.2) |
| Department | Pharmacy | 106 (60.9) |
|  | Medical Oncology | 21 (12.1) |
|  | Surgical Oncology | 14 (8.0) |
|  | Radiation Oncology | 11 (6.3) |
|  | ICU | 19 (10.9) |
|  | Other | 3 (1.7) |
| Years of Experience in Hospital Pharmacy | < 5 | 36 (20.7%) |
|  | 5 - 10 | 43(24.7%) |
|  | 11 - 20 | 62 (35.6%) |
|  | ＞20 | 33 (19.0%) |

Based on the results of the systematic review and cross-sectional survey, the multidisciplinary working group convened two structured consensus meetings to discuss the overall framework for the stratification of pharmaceutical care in cancer patients. The meetings focused on two key aspects: (1) the pharmaceutical care stratification for each indicator; (2) the "Anti-tumor Drug Toxicity Risk Management" section. Following extensive discussions, it was ultimately decided to classify toxicity by organ systems, which is elaborated in 10 separate sections, namely Cardiovascular System, Respiratory System, Digestive System, Endocrine System, Hematological System, Immune System, Urinary System, Nervous System, Skin and Mucosal System, and Other. Furthermore, the "Coagulation Function and Thrombosis/Bleeding History" indicator was removed, as it pertains to the complex field of the cardiovascular system. Cancer patients receiving anti-tumor drug therapy typically indicates that their cardiovascular system has already passed the relevant evaluations, and retaining this indicator would increase the complexity and inaccessibility in clinical practice. Additionally, the "Disease State" indicator was deleted, as it is already reflected by other existing indicators and does not provide unique value. Another indicator, "Smoking History," was removed due to its limited direct relevance to the stratification of cancer pharmaceutical care, with its potential impact adequately captured by other clinical assessment indicators. Moreover, the suggestions from the cross-sectional survey were specifically incorporated: two new indicators—psychological abnormalities and psychiatric disorders—were added, along with a "Complex Medication Issues" indicator for which a preliminary definition was formulated. The two consensus meetings ultimately finalized the questionnaire items for the subsequent expert consultation.

**3 Delphi Expert Consultation**

**3.1 Selection of Consulting Experts**

The Delphi method typically entails selecting 20-30 experts in the relevant research fields to conduct consultations via correspondence. The inclusion criteria for participating experts in this consensus are specified as follows: (1) holding professional qualifications as a pharmacist, physician, or evidence-based medicine specialist; (2) specializing in clinical pharmacy, clinical medicine, evidence-based medicine, or pharmacy administration; (3) possessing an associate senior professional title or higher; (4) holding a bachelor’s degree or above; (5) having accumulated at least 5 years of professional experience; and (6) demonstrating strong enthusiasm for the study and volunteering to participate.

**3.2 Expert Consultation Process**

After confirming the consulting experts, the multidisciplinary working group convened two structured consensus meetings. Drawing on the results of the systematic evidence review and nationwide practice survey, the group jointly drafted the preliminary framework of stratification indicators and core recommendations for pharmaceutical care. Through in-depth interdisciplinary discussions, the team refined the indicator definitions, classification logic, and applicability scope-resolving ambiguities and ensuring alignment with clinical practice needs and operational feasibility.

Subsequently, the research team developed the first-round consultation questionnaire based on the outcomes of these structured meetings and distributed it to the experts via WeChat and email in accordance with the Delphi method workflow. The team was responsible for questionnaire collection; based on the first-round survey results and expert feedback, some indicators were added, removed, or revised to form the second-round consultation questionnaire, which was then distributed for the second round of consultation. Following the two rounds of consultation, the importance scores of the items were re-evaluated. The consultation process was concluded if statistical analysis indicated that expert opinions had essentially converged.

The screening criteria for the consultation indicators in this study were set as follows: a mean importance score > 4 points and a coefficient of variation (CV) ≤ 20%. Indicators meeting both criteria were included. For those failing to meet one or both criteria, a decision was made after group discussions, taking into account clinical practice, professional characteristics, the inherent logical consistency of stratified management indicators, and expert consultation feedback.

**3.3 Statistical Analysis**

Microsoft Excel for mac software were used for data processing, and all statistical analyses were performed using R software (version 4.3.0, <https://www.R-project.org>). Continuous data were expressed as mean ± standard deviation (x̄ ± s) and analyzed using the t-test. Categorical data were presented as frequencies (n) and percentages (%), with the chi-square test (χ² test) applied for statistical analysis. The significance level (α) was set at 0.05.

**3.4 Results of Delphi Expert Consultation**

***3.4.1 Basic Characteristics of Consulting Experts***

From April 1 to June 30, 2025, the consensus panel conducted two rounds of round-table discussions via online meetings, followed by two rounds of expert consultations using questionnaires to solicit feedback. Details of the experts’ basic information are provided in Table S6.

**Table S6 Characteristics of the experts (N=28)**

| **Characters** | **Category** | **n (%)** |
| --- | --- | --- |
| Sex | Female | 15 (53.6) |
|  | Male | 13 (46.4) |
| Age, years | < 40 | 6 (21.4) |
|  | 40 - 50 | 10 (35.7 |
|  | ＞ 50 | 12 (42.9) |
| Type of Hospital | Tertary A | 27 (96.4) |
|  | Tertary B | 1 (3.6) |
|  | Secondary or Lower | 0 (0) |
| Highest Education Level | Bachelor's | 10 (35.7) |
|  | Master's | 12 (42.9) |
|  | PhD | 11 (39.3) |
| Academic Background | Pharmacy | 21 (75.0) |
|  | Clinical Medicine | 4 (14.3) |
|  | Evidence-Based Medicine | 2 (7.1) |
|  | Other | 1 (3.6) |
| Years of Experience in Hospital Pharmacy | < 10 | 0 (0) |
|  | 10 - 20 | 6 (21.4) |
|  | ＞20 | 22 (78.6) |

***3.4.2 Evaluation Results of the Expert Consultation Questionnaire***

The first round of consultation primarily included evaluations of the indicators’ definitions, categorization, importance ratings, feasibility ratings, experts’ basic information, and their familiarity with the consultation topics. The indicators were revised based on expert feedback to form the questionnaire for the second round. The second round of consultation mainly covered ratings of indicator importance, experts' basic information, and self-assessments of their “basis for judgment” and “familiarity”. Importance was rated using a Likert 5-point scale, ranging from “very important” to “unimportant” with scores assigned from 5 to 1 (1 being “very unimportant” and 5 “very important”).

All experts in this study completed both rounds of consultation and returned valid questionnaires, achieving a 100% response rate and validity rate for both rounds. In the first round, seven experts provided comments, which decreased to two in the second round. The mean expert authority coefficient (Cr) for both rounds was 0.952, as detailed in Table S7. The overall Kendall’s W coefficients for the two rounds were 0.290 and 0.242, respectively (with p-values from the χ² test both <0.001), indicating a high level of coordination and consensus among the experts. The results are shown in Table S8.

**Table S7 Experts' authority coefficients (Ca, Cs, Cr)**

| **Round** | **Ca** | **Cs** | **Cr** |
| --- | --- | --- | --- |
| 1 | 0.92±0.08 | 0.96±0.06 | 0.94±0.05 |
| 2 | 0.93±0.07 | 0.98±0.04 | 0.96±0.04 |

Note: Ca, coefficient of judgment basis; Cs, coefficient of familiarity; Cr, authority coefficient.

**Table S8 Statistical measures of expert consensus in two rounds**

| Round | Dimension | CV | χ² | Kendall's W | P-value |
| --- | --- | --- | --- | --- | --- |
| 1 | Knowledge | 0.201 | 570.780 | 0.755 | < 0.001 |
|  | Attitude | 0.163 | 591.948 | 0.783 | < 0.001 |
|  | Behavioral | 0.211 | 547.344 | 0.724 | < 0.001 |
| 2 | Knowledge | 0.125 | 555.128 | 0.862 | < 0.001 |
|  | Attitude | 0.136 | 544.180 | 0.845 | < 0.001 |
|  | Behavioral | 0.132 | 548.044 | 0.851 | < 0.001 |

Note: CV, Coefficient of Variation; χ², Chi-square value; df, Degree of Freedom (calculated as the number of items minus one)

**3.4.3 Indicator Revisions During Expert Consultation**

The first-round questionnaire included the definition and basic principles of stratified pharmaceutical care (3 items), implementation criteria (2 items), and 23 grading indicators. Based on predefined screening criteria and expert feedback, four indicators—those based on cancer staging, cardiac dysfunction, psychological abnormalities, and psychiatric disorders (Serial Numbers 10, 15, 17, and 18)—were removed. Additionally, the criterion "Agents for the first use in the medical institution" was added under "Antineoplastic Agents Toxicity Risk Management—Others" to address potential risks associated with newly introduced antineoplastic drugs.

In the second round, all indicators met the inclusion criteria. Two experts proposed revisions: one suggested refining the wording under "Dynamic Reassessment" for greater precision, which was accepted after discussion; the other recommended increasing the monitoring frequency to daily for Level 1 care and at least three times per week for Level 2 care. The working group declined the latter, considering it infeasible given current clinical pharmacist shortages in China. No further revisions were made. The working group concluded that consensus had been satisfactorily achieved and ended the consultation.

The final framework includes the definition, basic principles, implementation criteria, and 19 stratified pharmaceutical care indicators. Detailed item content, along with mean scores for importance, feasibility, and familiarity, are presented in Table S9.

**Table S9 Questionnaire on the Stratification System for Pharmaceutical Care in Cancer Patients**

**Basic Principles of Stratified Pharmaceutical Care**

| **No.** | **Items** | **Round 1** | | | **Round 2** | | |
| --- | --- | --- | --- | --- | --- | --- | --- |
|  |  | **Importance**  **(1-5)** | **Feasibility**  **(1-5)** | **Familiarity**  **(1-5)** | **Importance**  **(1-5)** | **Feasibility**  **(1-5)** | **Familiarity**  **(1-5)** |
|  |  |  |  |  |  |  |  |
| 1 | Pharmaceutical care for oncology patients is divided into three levels. | 4.64 ± 0.49 | 4.75 ± 0.52 | 4.79 ± 0.42 | 4.68 ± 0.61 | 4.71 ± 0.53 | 4.79 ± 0.50 |
| 2 | **Level 1 Pharmaceutical Care:** Applies to patients with life-threatening or potentially disabling conditions where pharmacotherapy critically influences survival or other key clinical outcomes. This level mandates high-intensity, real-time monitoring and proactive intervention by a pharmacist.  **Level 2 Pharmaceutical Care:** Indicated for severe or clinically significant conditions that are not immediately life-threatening but may significantly prolong hospitalization, increase costs, or compromise treatment adherence. It requires systematic evaluation, standardized monitoring, and active management of adverse events.  **Level 3 Pharmaceutical Care:** Designed for patients in a relatively stable disease state but with identifiable medication-related risks. The objective is to enhance quality of life and disease control through periodic review, patient education, and adherence support. | 4.25 ± 0.84 | 4.25 ± 0.75 | 4.32 ± 0.77 | 4.61 ± 0.74 | 4.71 ± 0.53 | 4.61 ± 0.50 |
| 3 | **Principles for Stratified Pharmaceutical Care**  **Inclusion Threshold:** Given the resource limitations in China, only patients classified as Level 3 or above are included in the pharmaceutical care scope.  **Highest Applicable Level:** When a patient meets the criteria for multiple levels, the highest applicable level of care is assigned.  **Dynamic Reassessment:** Any change in a patient's treatment or clinical status should trigger an immediate reassessment of the care level to ensure its continued appropriateness. | 4.21 ± 0.83 | 4.57 ± 0.69 | 4.18 ± 0.77 | 4.29 ± 0.76 | 4.82 ± 0.55 | 4.36 ± 0.73 |

**Implementation of Stratified Pharmaceutical Care**

| **No.** | **Items** | **Round 1** | | | **Round 2** | | |
| --- | --- | --- | --- | --- | --- | --- | --- |
|  |  | **Importance**  **(1-5)** | **Feasibility**  **(1-5)** | **Familiarity**  **(1-5)** | **Importance**  **(1-5)** | **Feasibility**  **(1-5)** | **Familiarity**  **(1-5)** |
|  |  |  |  |  |  |  |  |
| 4 | Pharmaceutical care implementation items: Order review, medication reconciliation, medication education, adjustment of diagnosis and treatment plans, evaluation of the rationality of antineoplastic regimens, monitoring of therapeutic efficacy, adverse drug reactions, drug treatment processes, medication adherence, and individualized medication monitoring. | 4.29 ± 0.8 | 4.64 ± 0.62 | 4.14 ± 0.76 | 4.25 ± 0.84 | 4.71 ± 0.4 | 4.18 ± 0.82 |
| **5** | **Level 1 Pharmaceutical Care Rounds / Frequency and Content:**   - **Frequency:** At least 3 times per week. - **Content:** Primarily involves order review, adjustment of diagnosis and treatment plans, evaluation of the rationality of antineoplastic regimens, monitoring of drug therapy, and individualized medication monitoring.   **Level 2 Pharmaceutical Care Rounds / Frequency and Content:**   - **Frequency:** At least once per week. - **Content:** Primarily involves order review, adjustment of diagnosis and treatment plans, evaluation of the rationality of antineoplastic regimens, and monitoring of drug therapy.   **Level 3 Pharmaceutical Care Rounds / Frequency and Content:**   - **Frequency:** At least once per week. - **Content:** Primarily involves order review and adjustment of diagnosis and treatment plans. | 4.04 ± 0.74 | 4.57 ± 0.63 | 4.32 ± 0.86 | 4.25 ± 0.70 | 4.54 ± 0.74 | 4.36 ± 0.78 |

**Stratification Criteria and Corresponding Content**

| **N0.** | **Items** | **Round 1** | | | **Round 2** | | |
| --- | --- | --- | --- | --- | --- | --- | --- |
|  |  | **Importance**  **(1-5)** | **Feasibility**  **(1-5)** | **Familiarity**  **(1-5)** | **Importance**  **(1-5)** | **Feasibility**  **(1-5)** | **Familiarity**  **(1-5)** |
|  |  |  |  |  |  |  |  |
|  | **Part 1 Pathophysiological Conditions** |  |  |  |  |  |  |
|  | **——Special Physiological Conditions** |  |  |  |  |  |  |
| 6 | Level 1 pharmaceutical care is recommended for cancer patients with fertility preservation needs scheduled for gonadotoxic therapy. | 4.11 ± 0.69 | 4.57 ± 0.5 | 4.21 ± 0.8 | 4.61 ± 0.69 | 4.79 ± 0.50 | 4.75 ± 0.59 |
| 7 | Level 3 pharmaceutical care is recommended for oncology patients aged ≥75 years. | 4.21 ± 0.83 | 4.71 ± 0.53 | 4.75 ± 0.52 | 4.39 ± 0.74 | 4.75 ± 0.4 | 4.79 ± 0.42 |
| 8 | Level 3 pharmaceutical care is recommended for cancer patients with underweight (BMI <18.5 kg/m²) or obesity (BMI ≥28 kg/m²). | 4.07 ± 0.77 | 4.71 ± 0.53 | 4.61 ± 0.63 | 4.14 ± 0.80 | 4.75 ± 0.44 | 4.64 ± 0.4 |
| 9 | Level 3 pharmaceutical care is recommended for cancer patients with an Eastern Cooperative Oncology Group (ECOG) performance status ≥2 or Karnofsky Performance Status (KPS) ≤70. | 4.07 ± 0.81 | 4.21 ± 0.69 | 4.18 ± 0.77 | 4.25 ± 0.84 | 4.29 ± 0.71 | 4.14 ± 0.80 |
|  | **——Special Pathological Conditions** |  |  |  |  |  |  |
| **10** | **Pharmaceutical care levels are assigned based on cancer staging:**   - Level 3 pharmaceutical care is recommended for patients with early-stage cancer. - Level 2 pharmaceutical care is recommended for patients with intermediate-stage or locally advanced cancer. - Level 1 pharmaceutical care is recommended for patients with advanced-stage cancer or systemic metastasis. | 3.50 ± 1.14 | 4.04 ± 0.92 | 4.11 ± 0.79 | / | / | / |
| 11 | Level 2 pharmaceutical care is recommended for cancer patients with unstable chronic comorbidities (e.g., hypertension, diabetes, chronic obstructive pulmonary disease [COPD]), whereas Level 3 care is recommended for those with relatively stable conditions. | 4.21 ± 0.79 | 4.11 ± 0.79 | 4.25 ± 0.75 | 4.18 ± 0.72 | 4.18 ± 0.77 | 4.54 ± 0.74 |
| 12 | For cancer patients with concurrent infections, stratified pharmaceutical care is recommended based on infection severity: Level 1 for severe infections, Level 2 for moderate infections, and Level 3 for mild infections. | 4.61 ± 0.63 | 4.57 ± 0.69 | 4.68 ± 0.48 | 4.75 ± 0.59 | 4.61 ± 0.63 | 4.71 ± 0.46 |
| 13 | ***Nutritional Status & Support***   - Level 2 pharmaceutical care is recommended for cancer patients with severe malnutrition (Patient-Generated Subjective Global Assessment [PG-SGA] grade C), and Level 3 care is recommended for those with suspected or mild malnutrition (PG-SGA grade B). - Level 3 pharmaceutical care is recommended for cancer patients with nutritional risk (Nutritional Risk Screening 2002 [NRS2002] score ≥3). - Level 3 pharmaceutical care is recommended for cancer patients receiving parenteral nutrition or enteral tube-feeding. | 4.04 ± 0.74 | 4.36 ± 0.68 | 4.18 ± 0.82 | 4.32 ± 0.7 | 4.25 ± 0.84 | 4.29 ± 0.85 |
| 14 | Level 2 pharmaceutical care is recommended for opioid-naive cancer patients, those with opioid-related safety risk factors (e.g., renal/hepatic dysfunction, respiratory impairment, sleep apnea, poor performance status), or those with inadequate pain control despite opioid use. | 4.36 ± 0.83 | 4.14 ± 0.76 | 4.21 ± 0.79 | 4.39 ± 0.83 | 4.29 ± 0.85 | 4.36 ± 0.68 |
| 15 | Stratified pharmaceutical care is recommended for cancer patients with cardiac dysfunction: Level 1 for severe dysfunction, Level 2 for moderate dysfunction, and Level 3 for mild dysfunction. | 3.29 ± 1.27 | 4.07 ± 0.81 | 4.25 ± 0.80 | / | / | / |
| 16 | Stratified pharmaceutical care is recommended for cancer patients with hepatic or renal impairment: Level 1 for severe impairment, Level 2 for moderate impairment, and Level 3 for mild impairment. | 4.61 ± 0.63 | 4.71 ± 0.53 | 4.57 ± 0.74 | 4.75 ± 0.52 | 4.71 ± 0.46 | 4.75 ± 0.59 |
| 17 | Level 2 pharmaceutical care is recommended for cancer patients with psychological abnormalities. | 2.68 ± 1.36 | 3.89 ± 0.83 | 3.50 ± 1.14 | / | / | / |
| 18 | Level 2 pharmaceutical care is recommended for cancer patients with psychiatric disorders requiring corresponding pharmacotherapy. | 2.96 ± 1.37 | 3.68 ± 0.67 | 3.36 ± 1.52 | / | / | / |
|  | **Part 2 Medication-Related Factors** |  |  |  |  |  |  |
|  | **——Antineoplastic Agents Toxicity Risk Management** |  |  |  |  |  |  |
| 19 | Stratified pharmaceutical care is recommended for cancer patients receiving potentially toxic antineoplastic agents, taking into account: (a) the agent’s toxicity profile (spectrum, incidence, severity, and reversibility); (b) patient-specific characteristics (comorbidities, age, and prior radiotherapy); and (c) complexity of clinical management (monitoring, prophylaxis, regimen adjustments, and prognosis). Including: cardiovascular system, respiratory system, digestive system, endocrine system, hematological system, immune system, urinary system, nervous system, skin and mucosal system and others. | 4.36 ± 0.83 | 4.07 ± 0.72 | 4.14 ± 0.80 | 4.57 ± 0.79 | 4.25 ± 0.84 | 4.18 ± 0.77 |
|  | **——Antineoplastic Agents Toxicity Management** |  |  |  |  |  |  |
| 20 | For cancer patients with adverse events (AEs) attributed to antineoplastic agents, stratified pharmaceutical care for drug-related toxicities is categorized into three levels in accordance with the Common Terminology Criteria for Adverse Events version 5.0 (CTCAE v5.0):   - Level 1 pharmaceutical care is implemented for life-threatening or disabling toxicities that necessitate permanent discontinuation of antineoplastic therapy. - Level 2 pharmaceutical care is implemented for patients experiencing severe or clinically significant toxicities that are not immediately life-threatening but may result in prolonged hospitalization, increased treatment costs, or reduced adherence. These toxicities often necessitate temporary interruption or dose adjustment of anticancer therapy, with treatment resumption allowed only after a comprehensive benefit–risk assessment. - Level 3 pharmaceutical care is implemented for all other antineoplastic-related toxicities that do not meet the criteria for Level 1 or Level 2. | 4.75 ± 0.44 | 4.29 ± 0.81 | 4.43 ± 0.69 | 4.82 ± 0.55 | 4.36 ± 0.83 | 4.61 ± 0.74 |
|  | **——Therapeutic Drug Monitoring (TDM) & Pharmacogenomics** |  |  |  |  |  |  |
| 21 | ***Therapeutic Drug Monitoring (TDM) & Pharmacogenomics***   - Level 1 pharmaceutical care is recommended for cancer patients with drug concentrations outside the target range and exhibiting toxicity. - Level 2 pharmaceutical care is recommended for cancer patients with concentrations within the target range but exhibiting toxicity, or outside the range without toxicity. - Level 3 pharmaceutical care is recommended for cancer patients with concentrations within the target range and without toxicity. - Level 2 pharmaceutical care is recommended for cancer patients with abnormal drug metabolism genotypes (ultra-rapid or poor metabolizers). | 4.18 ± 0.72 | 4.21 ± 0.74 | 4.32 ± 0.77 | 4.25 ± 0.80 | 4.18 ± 0.82 | 4.43 ± 0.69 |
|  | **——Concomitant Medication** |  |  |  |  |  |  |
| 22 | ***Clinically Significant Drug-Drug Interactions (DDIs)***  Level 2 pharmaceutical care is recommended for cancer patients with clinically significant drug–drug interactions (DDIs) that cannot be avoided, whereas Level 3 care is recommended when interactions are mitigated but still require monitoring. | 4.32 ± 0.72 | 4.18 ± 0.77 | 4.11 ± 0.74 | 4.50 ± 0.75 | 4.29 ± 0.71 | 4.36 ± 0.73 |
| 23 | Level 2 pharmaceutical care is recommended for cancer patients prescribed ≥10 non-antineoplastic medications, whereas Level 3 care is recommended for those prescribed 5–9 antineoplastic medications. | 4.32 ± 0.86 | 4.18 ± 0.77 | 4.21 ± 0.79 | 4.54 ± 0.79 | 4.14 ± 0.80 | 4.61 ± 0.57 |
|  | **——Special Routes of Administration or Delivery Devices** |  |  |  |  |  |  |
| 24 | Level 2 pharmaceutical care is recommended for cancer patients receiving enteral tube feeding with enteric-coated preparations, sustained-/controlled-release formulations, pharmaceutically incompatible medications, hyperosmolar fluids (>500 mOsm/L), or drugs prone to precipitation, as well as for those with gastrointestinal dysfunction requiring enteral feeding. | 4.07 ± 0.72 | 4.25 ± 0.84 | 4.18 ± 0.72 | 4.14 ± 0.80 | 4.32 ± 0.67 | 4.29 ± 0.85 |
|  | **——Medication Adherence** |  |  |  |  |  |  |
| 25 | Level 3 pharmaceutical care is recommended for cancer patients with suboptimal medication adherence (e.g., missed doses, incorrect administration, self-adjusted dosing, or treatment refusal). | 4.36 ± 0.73 | 4.68 ± 0.55 | 4.54 ± 0.58 | 4.29 ± 0.71 | 4.75 ± 0.52 | 4.57 ± 0.63 |
|  | **——Complex Medication Issues** |  |  |  |  |  |  |
| 26 | Level 2 pharmaceutical care is recommended for cancer patients with complex medication issues requiring collaborative decision-making by two or more clinical pharmacists from different specialties (e.g., oncology, anti-infectives, enteral/parenteral nutrition). | 4.18 ± 0.72 | 4.04 ± 0.79 | 4.21 ± 0.79 | 4.14 ± 0.71 | 4.18 ± 0.77 | 4.29 ± 0.81 |
|  | **Part 3 Non-Medication Therapeutic Interventions** |  |  |  |  |  |  |
| 27 | Level 1 pharmaceutical care is recommended for cancer patients experiencing life-threatening or disabling complications or AEs from non-medication treatments (e.g., radiotherapy, interventional therapy, or surgery); Level 2 care is recommended for severe events but non-life-threatening complications or AEs; and Level 3 care is recommended for mild-to-moderate complications or AEs. (When complications such as infection or hepatic/renal insufficiency occur, stratified care should be determined according to the corresponding criteria.) | 4.14 ± 0.80 | 4.11 ± 0.74 | 4.04 ± 0.79 | 4.32 ± 0.82 | 4.07 ± 0.81 | 4.04 ± 0.64 |
| 28 | Level 2 pharmaceutical care is recommended for cancer patients receiving immunocellular therapies (e.g., CAR-T, tumor-infiltrating lymphocyte [TIL]), with Level 1 care recommended for associated complications. For patients receiving gene therapy, targeted radionuclide therapy (e.g., ¹⁷⁷Lu-PSMA), or physical ablation/photodynamic therapy, Level 1 care is recommended for severe complications, and Level 2 care for mild-to-moderate complications. | 4.18 ± 0.67 | 4.04 ± 0.64 | 4.18 ± 0.77 | 4.14 ± 0.80 | 4.07 ± 0.81 | 4.21 ± 0.74 |

Reference

[1] Alshaikhmubarak FQ, Keers RN, Brown P, et al. Developing the Inpatient Mental Health Pharmaceutical Assessment and Care Tool (IMPACT) for use by UK mental health pharmacy teams—a modified Delphi study. Br J Clin Pharmacol, 2025: 91(10):2836-2853.

[2] Chen XF. Analysis of the application value of hierarchical pharmaceutical care in the clinical practice of diabetic patients. Contemporary Medical Symposium, 2024: 22(7):99-101. (in Chinese)

[3] Doublet S, Pagès A, Thomas ZA, et al. Systemic treatment among frail older patients with cancer: An observational cohort. J Geriatr Oncol, 2025: 16(2):102177.

[4] Huang JQ, Zhao GY, Shi CQ, et al. Exploration and practice of the pharmaceutical care work mode carried out by clinical pharmacists in the daytime oncology ward. China Prescription Drug, 2025: 23(18):53-57. (in Chinese)

[5] Luo W, Huang H, Zhou Y, et al. Implementation of pharmaceutical strategies using the PDCA cycle for standardized management of cancer pain medications. Support Care Cancer, 2025: 33(3):163.

[6] Mohammed AH, Hassan BAR, Sern LJ, et al. Development and validation of a questionnaire assessing pharmacists' knowledge and practice towards antimicrobial stewardship in oncology care. PLoS One, 2025: 20(5):e0321551.

[7] Muluneh B, Upchurch M, Mackler E, et al. Optimizing Adherence to Oral Anticancer Agents: Results from an Implementation Mapping Study. Curr Oncol, 2025: 32(2).

[8] Sun Y, Han NH. Establishment and effect evaluation of hierarchical pharmaceutical care pathway for children with epilepsy. Chinese Journal of Drugs and Clinical, 2025: 25(7):440-446. (in Chinese)

[9] Yang Y, Li Q, Zhou M. Practical Study on the Participation of Clinical Pharmacists in Multi-Disciplinary Teams Dealing With Nutrition Intervention Management in Ovarian Cancer. J Eval Clin Pract, 2025: 31(1):e14260.

[10] Bansal N, Campbell SM, Lin CY, et al. Development of prescribing indicators related to opioid-related harm in patients with chronic pain in primary care—a modified e-Delphi study. BMC Med, 2024: 22(1):5.

[11] Canning ML, Barras M, McDougall R, et al. Defining quality indicators, pharmaceutical care bundles and outcomes of clinical pharmacy service delivery using a Delphi consensus approach. Int J Clin Pharm, 2024: 46(2):451-462.

[12] Zhou Y, Cao XC, Yuan L. Application effect of hierarchical pharmaceutical care intervention in diabetic patients. Modern Health Preservation, 2024: 24(16):1252-1254. (in Chinese)

[13] de Souza JFF, Fernandes BD, Rotta I, et al. Key performance indicators for pharmaceutical services: A systematic review. Explor Res Clin Soc Pharm, 2024: 14:100441.

[14] Gossery C, Clarenne J, Barraud S, et al. Preliminary guidelines for the detection and management of drug-related problems in cancer patients with type 2 diabetes mellitus: a practical resource for oncology pharmacists. Support Care Cancer, 2024: 32(12):791.

[15] Lian YF, Qiu XJ, Yang JN, et al. Multi-dimensional value analysis of standardized hierarchical pharmaceutical care services in hospitalized patients with chronic airway diseases. China Pharmacy, 2024: 35(19):2404-2410. (in Chinese)

[16] Maezawa T, Yonemura M, Baba K, et al. Current Status and Issues of Collaboration Between Physicians and Pharmacists in Fertility Preservation. J Adolesc Young Adult Oncol, 2024: 13(3):514-522.

[17] Schönenberger N, Blanc AL, Hug BL, et al. Developing indicators for medication-related readmissions based on a Delphi consensus study. Res Social Adm Pharm, 2024: 20(6):92-101.

[18] Shrestha S, Iqbal A, Teoh SL, et al. Impact of pharmacist-delivered interventions on pain-related outcomes: An umbrella review of systematic reviews and meta-analyses. Res Social Adm Pharm, 2024: 20(6):34-51.

[19] Xue Z, Qi GX, Qi RR, et al. Practice and evaluation of clinical pharmacist-led anticoagulant therapy management in patients with atrial fibrillation. Clinical Rational Drug Use, 2024: 17(11):11-15. (in Chinese)

[20] Yu YF. Exploration of pharmaceutical care pathway for patients with depressive disorders and study on intervention effect. Hubei University of Medicine, 2024. (in Chinese)

[21] Zhang RB, Wu QH, Cao XR, et al. Construction of hierarchical management standards for patients with chronic obstructive pulmonary disease from the perspective of pharmacists based on the Delphi method. China Pharmacy, 2024: 35(7):860-865. (in Chinese)

[22] Zhao YP, Zhu XK, Da CL, et al. Effect evaluation of hierarchical pain management mode in perioperative patients with lung cancer. Gansu Medical Journal, 2024: 43(11):1023-1026. (in Chinese)

[23] Zhou H, Lü PF. Application of pharmaceutical extended services based on hierarchical assessment in elderly patients with chronic obstructive pulmonary disease. China Modern Medicine, 2024: 31(30):105-108+112. (in Chinese)

[24] Betts AC, Murphy CC, Shay LA, et al. Polypharmacy and prescription medication use in a population-based sample of adolescent and young adult cancer survivors. J Cancer Surviv, 2023: 17(4):1149-1160.

[25] Chang CE, Khan RA, Tay CY, et al. Development and validation of a pharmaceutical assessment screening tool to prioritise patient care in a tertiary care hospital. PLoS One, 2023: 18(3):e0282342.

[26] Chen JH. Clinical effect evaluation of hierarchical pharmaceutical care in diabetic patients. Chinese Science and Technology Journal Database (Abstract Edition) Medical and Health, 2023: (12):1-3. (in Chinese)

[27] Gong Y, Wei W, Zhang W, et al. Implementation effect of a hierarchical pharmaceutical service pattern in patients with systemic lupus erythematosus. J Int Med Res, 2023: 51(2):3000605231154749.

[28] Gong Y, Qi ZG, Huang Y, et al. Establishment and application of hierarchical pharmaceutical care pathway for patients with stroke-associated pneumonia. Pharmaceutical and Clinical Research, 2023: 31(2):168-171. (in Chinese)

[29] Jiang HH, Zhang QY, Huang Y, et al. Effect evaluation of standardized hierarchical pharmaceutical care in perioperative patients of urology department. Chinese Journal of Hospital Pharmacy, 2023: 43(22):2551-2557. (in Chinese)

[30] Jost N, Erickson N, Bratu E, et al. Closing the cancer care gap with a patient-reported nutrition screening: A retrospective analysis of a quality improvement project on an oncology ward (CCC study). Clin Nutr ESPEN, 2023: 57:246-252.

[31] Qin Q, Sheng WJ, Tang J, et al. Practice and clinical evaluation of hierarchical pharmaceutical care in diabetic patients. Herald of Medicine, 2023: 42(2):248-253. (in Chinese)

[32] Wang MM, Ma CL, Wen XY, et al. Pharmaceutical practice of clinical pharmacists in children with generalized myasthenia gravis. Pharmaceutical and Clinical Research, 2023: 31(6):543-546. (in Chinese)

[33] Wei W, Zhang W, Ding YL, et al. Practice and clinical evaluation of hierarchical pharmaceutical care in patients with heart failure. Chinese Journal of Hospital Pharmacy, 2023: 43(16):1856-1860. (in Chinese)

[34] Xie H, Chen X, Xue M, et al. Construction of a pharmaceutical care mode for cancer pain patients in primary care based on the Delphi method: an effective analysis. Front Pharmacol, 2023: 14:1268793.

[35] Zhang CG, Wu Q, Wei W, et al. Practice of hierarchical pharmaceutical service mode in patients with systemic lupus erythematosus. Chinese Pharmaceutical Journal, 2023: 58(6):537-543. (in Chinese)

[36] Bates N, Bello JK, Osazuwa-Peters N, et al. Depression and Long-Term Prescription Opioid Use and Opioid Use Disorder: Implications for Pain Management in Cancer. Curr Treat Options Oncol, 2022: 23(3):348-358.

[37] Botelho SF, Pantuzza LLN, Marinho CP, et al. Consensus on the criteria for patient prioritization in hospital clinical pharmacy services: a Delphi study. Int J Clin Pharm, 2022: 44(4):985-992.

[38] Chai DY, Lao HY, Zhao CL, et al. Discussion on hierarchical services of medication reconciliation based on work practice. Pharmacy Today, 2022: 32(5):392-396. (in Chinese)

[39] Chen Y, Li SY, Xu LL, et al. Application effect of Triangle hierarchical management model in the management of patients with dual antiplatelet therapy. Chinese Journal of Clinical Pharmacy, 2022: 31(11):841-845. (in Chinese)

[40] Dai MF, Li SY, Wang BY, et al. Establishment and practice of hierarchical management mode for warfarin anticoagulant patients based on Triangle theory. Chinese Pharmaceutical Journal, 2022: 57(21):1800-1804. (in Chinese)

[41] En-Nasery-de Heer S, Tromp V, Westerman MJ, et al. Patient experiences and views on pharmaceutical care during adjuvant endocrine therapy for breast cancer: A qualitative study. Eur J Cancer Care (Engl), 2022: 31(6):e13749.

[42] Fang Y, Chen N, Ge CL, et al. Effect evaluation of hierarchical pharmaceutical care provided by multidisciplinary pharmacist team for patients undergoing complex gastrointestinal surgery in general surgery department. Herald of Medicine, 2022: 41(10):1460-1463. (in Chinese)

[43] He PH, Xu FL, Ruan SF, et al. Discussion on the application of hierarchical pharmaceutical care mode in patients with schizophrenia. Pharmacy Today, 2022: 32(2):142-145+149. (in Chinese)

[44] Hoegy D, Martin J, Barral M, et al. Development of clinical pharmacy programs integrated into patient care pathways using adverse event risks. Res Social Adm Pharm, 2022: 18(6):3052-3057.

[45] Jiang J, Zhang YF, Wang JL, et al. Practical effect of hierarchical management and classification evaluation system of pharmaceutical care in ICU. Chinese Journal of Clinical Pharmacy, 2022: 31(8):577-583. (in Chinese)

[46] Lockman K, Lowry MF, DiScala S, et al. Development of Entrustable Professional Activities for Specialist Hospice and Palliative Care Pharmacists. J Pain Symptom Manage, 2022: 64(1):37-48.

[47] Mashni OK, Nazer LH, Khalil HZ, et al. Impact of Clinical Pharmacy Services on Patient Management in the Chemotherapy Infusion Clinics: A 5-Year Study at a Comprehensive Cancer Center. J Pharm Pract, 2022: 35(5):686-690.

[48] Chinese Society of Clinical Oncology. China guideline for pharmaceutical care on anti-tumor antibody-based drugs. Chinese Journal of Oncology, 2022: 44(10):1017-1046. (in Chinese)

[49] Pirolli AV, Brusamarello T, Everton SS, et al. The role of the clinical pharmacist in guiding adjuvant hormonal therapy in patients with breast cancer. J Oncol Pharm Pract, 2022: 28(6):1368-1374.

[50] Sato N, Fujita K, Kushida K, et al. Development and consensus testing of quality indicators for geriatric pharmacotherapy in primary care using a modified Delphi study. Int J Clin Pharm, 2022: 44(2):517-538.

[51] Shawahna R, Shraim N, Aqel R. Views, knowledge, and practices of hospital pharmacists about using clinical pharmacokinetics to optimize pharmaceutical care services: a cross-sectional study. BMC Health Serv Res, 2022: 22(1):411.

[52] Wang LM, Li SY, Chen Y, et al. Efficiency evaluation of warfarin anticoagulant hierarchical management mode based on DEA method. Chinese Journal of Hospital Pharmacy, 2022: 42(24):2665-2669. (in Chinese)

[53] Li SY, Wang BY, Ge WH, et al. Practice and exploration of pharmaceutical mode of hierarchical management in anticoagulant clinic. Pharmaceutical and Clinical Research, 2021: 29(1):65-68. (in Chinese)

[54] Moukafih B, Abahssain H, Mrabti H, et al. Impact of clinical pharmacy services in a hematology/oncology ward in Morocco. J Oncol Pharm Pract, 2021: 27(2):305-311.

[55] Bakker T, Klopotowska JE, de Keizer NF, et al. Improving medication safety in the Intensive Care by identifying relevant drug-drug interactions - Results of a multicenter Delphi study. J Crit Care, 2020: 57:134-140.

[56] Qiu JR, Li D, Ding DM. Analysis of the implementation of hierarchical medication therapy management in pharmaceutical outpatient clinic. Chinese Science and Technology Journal Database (Full Text Edition) Medical and Health, 2020: (11):251-251+253. (in Chinese)

[57] Chen M, Qin K, Shen J, et al. Evaluation of indicator system of nutrition support pharmaceutical care mode using Delphi method. Chinese Journal of Hospital Pharmacy, 2020: 40(22):2366-2371. (in Chinese)

[58] Pamulapati LG, Rochester-Eyeguokan CD, Pincus KJ. Best practices for safe use of SGLT-2 inhibitors developed from an expert panel Delphi consensus process. Am J Health Syst Pharm, 2020: 77(21):1727-1738.

[59] Przybylski DJ, Dow-Hillgartner EN, Reed MP, et al. Current state assessment survey of challenges of pharmacogenomics within oncology pharmacy practice. J Oncol Pharm Pract, 2020: 26(6):1374-1381.

[60] Shawahna R. Quality Indicators of Pharmaceutical Care for Integrative Healthcare: A Scoping Review of Indicators Developed Using the Delphi Technique. Evid Based Complement Alternat Med, 2020: 2020:9131850.

[61] Vu K, Pardhan A, Lakhani N, et al. Managing chemotherapy-related toxicities in the community setting: A survey of pharmacists in Ontario. J Oncol Pharm Pract, 2020: 26(8):1903-1911.

[62] Boşnak AS, Birand N, Diker Ö, et al. The role of the pharmacist in the multidisciplinary approach to the prevention and resolution of drug-related problems in cancer chemotherapy. J Oncol Pharm Pract, 2019: 25(6):1312-1320.

[63] Boutin K, Nevers W, Gorman SK, et al. Development of intervention-related quality indicators for renal clinical pharmacists using a modified Delphi approach. Int J Pharm Pract, 2019: 27(5):436-442.

[64] Hou JQ, Zhang HM, Fu XJ, et al. Exploration and effect evaluation of hierarchical pharmaceutical care mode in COPD patients. Practical Pharmacy and Clinical Remedies, 2019: 22(2):182-185. (in Chinese)

[65] Hu WJ, Zeng N, Sun HJ, et al. Study on a new mode of standardized pharmaceutical management for children with asthma based on hierarchical management system of asthma symptom control level. Journal of Pediatric Pharmacy, 2019: 25(6):34-38. (in Chinese)

[66] McGrattan M, Barry HE, Ryan C, et al. The development of a Core Outcome Set for medicines management interventions for people with dementia in primary care. Age Ageing, 2019: 48(2):260-266.

[67] Mubarak N, Hatah E, Aris MAM, et al. Consensus among healthcare stakeholders on a collaborative medication therapy management model for chronic diseases in Malaysia; A Delphi study. PLoS One, 2019: 14(5):e0216563.

[68] Patel H, Aguiar PM, Pessoa A Jr, et al. Identifying quality of life indicators to improve outpatient pharmacy services for prostate cancer patients: a comparison between Brazilian and British experiences. Int Braz J Urol, 2019: 45(3):435-448.

[69] Qin Q, Chen R, Zhang Y, et al. Implementation and clinical evaluation of hierarchical pharmaceutical care in patients with asthma and chronic obstructive pulmonary disease. Chinese Pharmaceutical Journal, 2017: 52(16):1460-1464. (in Chinese)

[70] Zeh S, Christalle E, Hahlweg P, et al. Assessing the relevance and implementation of patient-centredness from the patients' perspective in Germany: results of a Delphi study. BMJ Open, 2019: 9(12):e031741.

[71] Zhang YL, Wang Q, Shang YG, et al. Hierarchical pharmaceutical care for 1 patient with postoperative infection in neurosurgery department. Strait Pharmaceutical Journal, 2017: 29(6):228-230. (in Chinese)

[72] Zuo J, Li HS. Discussion on the connotation and hierarchical system of pharmaceutical care based on resource cost. China Pharmaceuticals, 2019: 28(16):73-76. (in Chinese)

[73] Cheng K. Clinical significance of precise hierarchical pharmaceutical care in patients with chronic obstructive pulmonary disease. China Prescription Drug, 2018: 16(12):57-58. (in Chinese)

[74] Tian WW, Liu X, Ma MH, et al. Construction of anticoagulant hierarchical management service mode. Chinese Journal of Clinical Pharmacy, 2018: 27(2):130-132. (in Chinese)

[75] Watanabe T, Mikami M, Katabuchi H, et al. Quality indicators for cervical cancer care in Japan. J Gynecol Oncol, 2018: 29(6):e83.

[76] Colombo LRP, Aguiar PM, Lima TM, et al. The effects of pharmacist interventions on adult outpatients with cancer: A systematic review. J Clin Pharm Ther, 2017: 42(4):414-424.

[77] Gavila J, Seguí M, Calvo L, et al. Evaluation and management of chemotherapy-induced cardiotoxicity in breast cancer: a Delphi study. Clin Transl Oncol, 2017: 19(1):91-104.

[78] Moulin SM, Eutrópio FJ, Souza JO, et al. The role of clinical pharmacists in treatment adherence: fast impact in suppression of chronic myeloid leukemia development and symptoms. Support Care Cancer, 2017: 25(3):951-955.

[79] Shen XY, Fang Y, Zhu DQ. Establishment of hierarchical pharmaceutical care file for patients in cardiac intensive care unit. Shanghai Medical and Pharmaceutical Journal, 2016: 37(21):54-56. (in Chinese)

[80] Terada T. Pharmaceutical Investigation for Individualized and Optimal Cancer Pharmacotherapy. Yakugaku Zasshi, 2016: 136(11):1469-1476.

[81] Bu YS, Xu YG, Chen F, et al. Discussion on the formulation and implementation of hierarchical pharmaceutical care. Chinese Journal of Hospital Pharmacy, 2015: 35(24):2163-2165. (in Chinese)

[82] Gao N, Zhang ZJ, Wu DY, et al. Hierarchical pharmaceutical care and role of clinical pharmacists in medical oncology treatment. In: Proceedings of the 2015 Chinese Clinical Pharmacy Annual Conference and the 11th Chinese Clinical Pharmacist Forum. 2015. (in Chinese)

[83] Mahabaleshwarkar R, Khanna R, Banahan B, et al. Impact of Preexisting Mental Illnesses on Receipt of Guideline-Consistent Breast Cancer Treatment and Health Care Utilization. Popul Health Manag, 2015: 18(6):449-458.

[84] Dreesen M, Foulon V, Hiele M, et al. Quality of care for cancer patients on home parenteral nutrition: development of key interventions and outcome indicators using a two-round Delphi approach. Support Care Cancer, 2013: 21(5):1373-1381.

[85] Fiß T, Thyrian JR, Wucherer D, et al. Medication management for people with dementia in primary care: description of implementation in the DelpHi study. BMC Geriatr, 2013: 13:121.

[86] Ross LA, Bloodworth LS. Patient-centered health care using pharmacist-delivered medication therapy management in rural Mississippi. J Am Pharm Assoc (2003), 2012: 52(6):802-809.

[87] Wierenga PC, Klopotowska JE, Smorenburg SM, et al. Quality indicators for in-hospital pharmaceutical care of Dutch elderly patients: development and validation of an ACOVE-based quality indicator set. Drugs Aging, 2011: 28(4):295-304.

[88] Lai PS, Chua SS, Chew YY, et al. Effects of pharmaceutical care on adherence and persistence to bisphosphonates in postmenopausal osteoporotic women. J Clin Pharm Ther, 2011: 36(5):557-567.

[89] Chan A, Tan SH, Wong CM, et al. Clinically significant drug-drug interactions between oral anticancer agents and nonanticancer agents: a Delphi survey of oncology pharmacists. Clin Ther, 2009: 31(Pt 2):2379-2386.

[90] Scarpace SL, Brodzik FA, Mehdi S, et al. Treatment of head and neck cancers: issues for clinical pharmacists. Pharmacotherapy, 2009: 29(5):578-592.

[91] Setoguchi S, Earle CC, Glynn R, et al. Comparison of prospective and retrospective indicators of the quality of end-of-life cancer care. J Clin Oncol, 2008: 26(35):5671-5678.

[92] Hayakawa T, Yamanouchi K, Hirohata T, et al. Establishment of pharmaceutical management for patient care during lung cancer chemotherapy and its quantitative evaluation. Yakugaku Zasshi, 2005: 125(5):405-416.

[93] Hermansen-Kobulnicky CJ, Wiederholt JB, Chewning B. Adverse effect monitoring: opportunity for patient care and pharmacy practice. J Am Pharm Assoc (2003), 2004: 44(1):75-86; quiz 87-88.

[94] Edington J, Winter PD, Coles SJ, et al. Outcomes of undernutrition in patients in the community with cancer or cardiovascular disease. Proc Nutr Soc, 1999: 58(3):655-661.
