# Supplementary material for: Stratification system for pharmaceutical care in cancer patients: Chinese expert consensus
Source: Front Pharmacol. 2026 Feb 16;16:1707229. doi: 10.3389/fphar.2025.1707229 (PMC12950709; doi:10.3389/fphar.2025.1707229)
Supplement: Supplementary file 2 [file Supplementaryfile3.docx]

**Supplementary Material 3**

**List of Acknowledged Experts**

This list acknowledges the experts who made exceptional contributions to the development of this consensus.

| **Name** | **Affiliation** |
| --- | --- |
| Bikui Zhang | The Second Xiangya Hospital of Central South University |
| Bo Zhang | Peking Union Medical College Hospital |
| Chen Shi | Union Hospital, Tongji Medical College, Huazhong University of Science and Technology |
| Dong Liu | Tongji Hospital, Tongji Medical College, Huazhong University of Science and Technology |
| Haibin Dai | The Second Affiliated Hospital of Zhejiang University School of Medicine |
| Hejing Cao | Airport Hospital of Tianjin Cancer Institute & Hospital |
| Jiajie Luan | The First Affiliated Hospital of Wannan Medical College |
| Jianguo Zhu | The First Affiliated Hospital of Soochow University |
| Jie Zhang | Tianjin Medical University Cancer Institute & Hospital |
| Jifu Wei | Department of Pharmacy, Jiangsu Cancer Hospital |
| Jingwen Wang | Department of Pharmacy, Xijing Hospital |
| Jinhan He | West China Hospital, Sichuan University |
| Jinqi Li | Sichuan Provincial People's Hospital |
| Jiyong Liu | Fudan University Shanghai Cancer Center |
| Junshan Ruan | Fujian Provincial Hospital |
| Junyan Wu | Sun Yat-sen Memorial Hospital, Sun Yat-sen University |
| Kejing Tang | The First Affiliated Hospital, Sun Yat-sen University |
| Liqin Tang | The First Affiliated Hospital of University of Science and Technology of China (Anhui Provincial Hospital) |
| Luo Fang | Zhejiang Cancer Hospital |
| Manman Fu | Northeast International Hospital |
| Maobai Liu | Fujian Medical University Union Hospital |
| Minghui Long | Hunan Cancer Hospital |
| Peixi Zhao | Shanxi Cancer Hospital |
| Qian Jiang | Sichuan Cancer Hospital |
| Qingwei Zhao | The First Affiliated Hospital of Zhejiang University School of Medicine |
| Shao Liu | Xiangya Hospital, Central South University |
| Shujia Kong | Department of Pharmacy, Yunnan Cancer Hospital |
| Tao Liu | Sun Yat-sen University Cancer Center |
| Wanyi Chen | Chongqing University Cancer Hospital |
| Weiyi Feng | The First Affiliated Hospital of Xi'an Jiaotong University |
| Xiaoyan Dai | Department of Pharmacy, Gansu Cancer Hospital |
| Xiaoyu Li | Zhongshan Hospital, Fudan University |
| Xin Hai | The First Affiliated Hospital of Harbin Medical University |
| Yanhua Zhang | Peking University Cancer Hospital |
| Yanqing Song | The First Hospital of Jilin University |
| Ying Zhou | Peking University First Hospital |
| Youhong Hu | The First Affiliated Hospital of Zhengzhou University |
| Yuguo Liu | Shandong Cancer Hospital |
| Zhaohui Jin | West China Hospital, Sichuan University |
| Zhiying Hao | Shanxi Cancer Hospital |

Note: This list is sorted alphabetically by the experts' last names.
