# Supplementary material for: Stratification system for pharmaceutical care in cancer patients: Chinese expert consensus
Source: Front Pharmacol. 2026 Feb 16;16:1707229. doi: 10.3389/fphar.2025.1707229 (PMC12950709; doi:10.3389/fphar.2025.1707229)
Supplement: Supplementary file 3 [file Supplementaryfile2.docx]

**Supplementary Material 2**

**Risk of infertility associated with antineoplastic treatment in adult cancer patients ^#^.**

| **Cancer treatment** | **Fertility related outcome** | |
| --- | --- | --- |
|  | **Women** | **Men** |
| AC x 4 in < 40 years | Amenorrhea | Not applicable |
| AC - T in < 40 years | Amenorrhea | Not applicable |
| FEC, FAC in < 30 years | Amenorrhea | Not applicable |
| Tamoxifen | Decreased pregnancy/Amenorrhea | Not applicable |
| Trastuzumab | Amenorrhea | Not applicable |
| BEP | Decreased pregnancy/Amenorrhea | Decreased pregnancy (sired) |
| CHOP | Amenorrhea | Azoospermia |
| Methotrexate | Amenorrhea | Azoospermia |
| ABVD | Decreased pregnancy/Amenorrhea | Azoospermia |
| Actinomycin D | Amenorrhea | Azoospermia* |
| FOLFOX | Amenorrhea | Unknown |
| AC x 4 in ≥ 40 years | Amenorrhea | Not applicable |
| AC - T in ≥ 40 years | Amenorrhea | Not applicable |
| FEC, FAC in 30 - 39 years | Amenorrhea | Not applicable |
| TC、TAC | Amenorrhea | Not applicable |
| Carboplatin | Amenorrhea | Azoospermia |
| Cisplatin < 600 mg/m² | Amenorrhea | Azoospermia |
| BEACOPP | Amenorrhea | Azoospermia |
| Ifosfamide | Amenorrhea | Azoospermia* |
| Cisplatin > 600 mg/m² | Amenorrhea | Azoospermia |
| HCT conditioning (CT or TBI) | Decreased pregnancy/gonadal insufficiency | Decreased pregnancy (sired)/gonadal insufficiency |
| Pelvic radiation | Decreased pregnancy/gonadal insufficiency | Decreased pregnancy (sired)/gonadal insufficiency |

Low risk (<25% decrease in likelihood of pregnancy/fertility or <25% increase in risk of infertility)

High risk (>75% decrease in likelihood of pregnancy/fertility or >75% increase in risk of infertility)

# Santaballa A, Márquez-Vega C, Rodríguez-Lescure Á, et al. Multidisciplinary consensus on the criteria for fertility preservation in cancer patients. Clin Transl Oncol, 2022: 24(2):227-243.

*Azoospermia likely when given with other highly sterilizing agents. *ABVD* doxorubicin (Adriamycin^®^), bleomycin, vinblastine and dacarbazine; *AC* doxorubicin (Adriamycin^®^) and cyclophosphamide; *AC-T* doxorubicin (Adriamycin^®^), cyclophosphamide and paclitaxel (Taxol^®^); *BEACOPP* bleomycin, etoposide, doxorubicin (Adriamycin^®^), cyclophosphamide, vincristine (Oncovin^®^), procarbazine and prednisone; *BEP* bleomycin, etoposide and platinum; *CHOP* cyclophosphamide, doxorubicin (Adriamycin^®^), vincristine and prednisone; *CT* chemotherapy; *FAC* fuorouracil, doxorubicin (Adriamycin^®^) and cyclophosphamide; *FEC* fuorouracil, epirubicin and cyclophosphamide; *FOLFOX* fuorouracil, leucovorin and oxaliplatin; *HCT* haematopoietic cell transplantation; *TAC* docetaxel (Taxotere^®^), doxorubicin (Adriamycin^®^) and cyclophosphamide; *TBI* total body irradiation; *TC* docetaxel (Taxotere^®^) and cyclophosphamide.
